# Effects of Commercial Apple Varieties on Human Gut Microbiota Composition and Metabolic Output Using an In Vitro Colonic Model

**DOI:** 10.3390/nu9060533

**Published:** 2017-05-24

**Authors:** Athanasios Koutsos, Maria Lima, Lorenza Conterno, Mattia Gasperotti, Martina Bianchi, Francesca Fava, Urska Vrhovsek, Julie A. Lovegrove, Kieran M. Tuohy

**Affiliations:** 1 Hugh Sinclair Unit of Human Nutrition and the Institute for Cardiovascular and Metabolic Research (ICMR), Department of Food and Nutritional Sciences, University of Reading, Whiteknights, P.O. Box 226, Reading RG6 6AP, UK; j.a.lovegrove@reading.ac.uk; 2 Nutrition & Nutrigenomics Unit, Department of Food Quality and Nutrition, Research and Innovation Centre, Fondazione Edmund Mach (FEM), Via Mach 1, 38010 San Michele all’Adige, TN, Italy; Lima80in@yahoo.co.in (M.L.); lorenza.conterno@oliocru.it (L.C.); martina.bianchi.1@gmail.com (M.B.); francesca.fava@fmach.it (F.F.); kieran.tuohy@fmach.it (K.M.T.); 3 Metabolomics Unit, Department of Food Quality and Nutrition, Research and Innovation Centre, Fondazione Edmund Mach (FEM), Via Mach 1, 38010 San Michele all’Adige, TN, Italy; mattiagasperotti84@gmail.com (M.G.); urska.vrhovsek@fmach.it (U.V.)

**Keywords:** apples, polyphenols, proanthocyanidins, fiber, pectin, gut microbiota, in vitro batch culture fermentation, microbial metabolites, Illumina 16S rRNA gene sequencing, Fluorescence in situ hybridization (FISH)

## Abstract

Apples are a rich source of polyphenols and fiber. A major proportion of apple polyphenols escape absorption in the small intestine and together with non-digestible polysaccharides reach the colon, where they can serve as substrates for bacterial fermentation. Animal studies suggest a synergistic interaction between apple polyphenols and the soluble fiber pectin; however, the effects of whole apples on human gut microbiota are less extensively studied. Three commercial apple varieties—Renetta Canada, Golden Delicious and Pink Lady—were digested and fermented in vitro using a batch culture colonic model (pH 5.5–6.0, 37 °C) inoculated with feces from three healthy donors. Inulin and cellulose were used as a readily and a poorly fermentable plant fiber, respectively. Fecal microbiota composition was measured by 16S rRNA gene Illumina MiSeq sequencing (V3-V4 region) and Fluorescence in Situ Hybridization. Short chain fatty acids (SCFAs) and polyphenol microbial metabolites were determined. The three apple varieties significantly changed bacterial diversity, increased Actinobacteria relative abundance, acetate, propionate and total SCFAs (*p* < 0.05). Renetta Canada and Golden Delicious significantly decreased Bacteroidetes abundance and increased Proteobacteria proportion and bifidobacteria population (*p* < 0.05). Renetta Canada also increased *Faecalibacterium prausnitzii*, butyrate levels and polyphenol microbial metabolites (*p* < 0.05). Together, these data suggest that apples, particularly Renetta Canada, can induce substantial changes in microbiota composition and metabolic activity in vitro, which could be associated with potential benefits to human health. Human intervention studies are necessary to confirm these data and potential beneficial effects.

## 1. Introduction

Evidence suggests that plant-derived dietary polyphenols and fiber possess health-promoting properties [1]. Apples are among the most popular and frequently consumed fruits in the world and a rich source of both polyphenols and fiber [2]. Epidemiological and dietary intervention studies suggest that frequent apple consumption is associated with a reduced risk of chronic pathologies such as cardiovascular disease, obesity and cancer [2,3]. However, up to 90–95% of dietary polyphenols are not absorbed in the small intestine [4] and together with non-digestible polysaccharides from apples reach the colon almost intact, where they can interact with the gut microbiota [2]. This interaction is reciprocal. Firstly, polyphenols and fiber undergo an extensive microbial bioconversion producing phenolic acids and short chain fatty acids (SCFAs), respectively, well-known to have positive health effects [5]. Secondly, polyphenols, fiber and/or their metabolic products modulate the gut microbiota composition by inhibiting pathogenic bacteria and stimulating beneficial bacteria, therefore acting as potential prebiotics [5].

Dietary fiber constituents in apples include insoluble fiber, mainly cellulose and hemicellulose and soluble fiber, mainly pectin. In vitro studies, using human fecal inoculum, have shown that pectin is fermented to SCFAs (acetate, propionate and butyrate) by several intestinal bacteria genera including *Bacteroides*, eubacteria, clostridia and bifidobacteria [6,7]. However, a recent study suggested a high selectivity at the species level [8]. Apples also contain a variety of polyphenols including dihydrochalcones, flavonols, hydroxycinnamates and flavanols (catechin and proanthocyanidins (PAs)) [9]. PAs, the major polyphenolic class in apples, also known as condensed tannins, are oligomers and polymers of flavanols and the most likely to reach the colon [2]. In an in vitro study, apple PAs have been shown to be converted to polyphenol microbial metabolites, mainly phenylpropionic, phenylacetic and benzoic acid derivatives. [10]. The study also reported reduced saccharolytic fermentation, suggesting potential antimicrobial properties of PAs. However, the specific bacteria composition was not explored [10].

In vivo, extraction juices from apple pomace, rich in polyphenols and fiber, have been shown to increase *Lactobacillus*, *Bifidobacterium, Bacteroidaceae* species, *Eubacterium rectale* cluster as well as SCFAs in rats [11,12]. Licht et al. (2010) [13] considered pectin, among apple components, responsible for a decrease in *Bacteroides* spp. and an increase in *Clostridium coccoides* and butyrate in rats [13]. Likewise, apple pectin restored the Firmicutes/Bacteroidetes ratio to normal in obesity-induced rats [14]. Decreased Firmicutes/Bacteroidetes ratio was similarly seen in mice with the administration of apple PAs, which also increased the proportion of *Akkermansia* [15]. However, a rat cecum fermentation showed that apple polyphenols and pectin are more effective in combination implying a synergistic effect [16]. Data available from human studies are still limited [2]. In a small scale trial of eight people, two apples per day for two weeks significantly increased bifidobacteria while reducing *Enterobacteriaceae* and lecithinace-positive clostridia, including *C. perfringens* [17]. In a more recent four-week study of 23 healthy subjects, whole apple and pomace intake lowered fecal pH but there were no changes in gut microbiota composition [18].

Thus, to date, the effects of apple components on gut microbiota have been explored mainly in animals and using extracted juices [11,12], PAs [15] or pectin [14] alone. There are no in vitro studies investigating the effects of whole apples using human fecal inoculum and only recently studies with apple components have focused on the entire gut community instead of targeted taxa [13,15]. The aim of the current work was to assess the effect of three commercial apple varieties—Renetta Canada, Golden Delicious and Pink Lady—on human gut microbiota composition and metabolic activity in vitro, compared to inulin (a prebiotic) and cellulose (poorly fermented). Illumina 16S rRNA sequencing was used to provide a broad picture of the microbial community architecture. Bacteria of specific interest (i.e., bifidobacteria and *Faecalibacterium prausnitzii*) were enumerated using the quantitative 16S rRNA probe based method, FISH. The production of SCFAs (acetate, propionate and butyrate) was also measured. Finally, disappearance of apple polyphenols and formation of microbial-derived metabolites were monitored throughout the fermentation using a targeted LC-MS based metabolomics approach.

## 2. Materials and Methods

### 2.1. Fecal Donors

Fecal donors, two males and one female, were in good health and aged between 30 and 50. They had not received antibiotic treatment for at least 3 months prior to stool collection, had not knowingly consumed pre- or probiotic supplements prior to experiment, and had no history of bowel disorders. The three healthy donors were informed of the study aims and procedures and provided their verbal consent for their fecal matter to be used for the experiments, in compliance with the ethics procedures required at the University of Reading and Fondazione Edmund Mach.

### 2.2. Materials

Enzymes for the apples digestion and chemicals for the batch culture basal nutrient medium were purchased from Sigma-Aldrich (St. Louis, MO, USA) and Applichem (Darmstadt, Germany), unless otherwise stated. For the chemical standards of polyphenols and microbial metabolites as well as the LC-MS reagents more information can be found in Vrhovsek et al. (2012) [19] and Gasperotti et al. (2014) [20].

### 2.3. Apples and Controls

The three commercial apple varieties, Renetta Canada, Golden Delicious and Pink Lady were purchased from a local shop in the Trentino region in north Italy. The apples’ macronutrient composition was analyzed by Campden BRI laboratories, UK, whereas the detailed polyphenol content was measured in our laboratory in Fondazione Edmund Mach based on Vrhovsek et al. (2012) [19]. Inulin (from dahlia tubers) and cellulose were used as a readily and a poorly fermentable plant fiber, respectively.

### 2.4. Preparation of Phospholipid Vesicles

A protocol was followed according to Mandalari et al. (2008) [21], with some modifications, for the preparation of the phospholipid vesicles and the simulation of the in vitro gastric and duodenal digestion as described below. Egg l-α-phosphatidylcholine (PC, lecithin grade 1, 99% purity, Lipid Products, Surrey, UK), 6.5 mL of a stock solution (127 mmol/L in chloroform/methanol), was placed into a round-bottom flask, and dried under rotary evaporation to make a thin phospholipid film. The lipid film was further dried overnight under vacuum to remove any remaining solvent. Then, it was hydrated with the addition of 170 mL of warm saline (150 mmol/L NaCl, pH 2.5, at 37 °C). The flask was flushed with argon to prevent oxidation and was placed in an orbital shaker (170 rpm, 37 °C) for 30 min together with five 2 mm diameter glass beads. A PC nanoemulsion was then produced using a Branson Ultrasonics sonifier S-450 (Branson Ultrasonics, Danbury, CT, USA) equipped with a 13 mm titanium horn (30% of amplitude). The temperature of the liquid kept below 60 °C with ice.

### 2.5. In Vitro Gastric and Duodenal Digestion

A ratio of 4 g of apples for 12.4 mL gastric phase volume (acidic saline) considered appropriate after preliminary experiments and according to Mandalari et al. (2008) [21]. Initially, 96 g of each apple variety were grated with their skin and added to 146 mL of the sonicated and filtrated PC vesicle suspension. The pH was adjusted to 2.5 using HCl and acidic saline (150 mmol/L NaCl, pH 2.5) was added to a total volume of 298 mL. Then, the PC vesicle suspension together with gastric pepsin and lipase were added so that the final concentrations were 2.4 mmol/L, 146 units/mL and 60 units/mL, respectively. The digestion was performed in an orbital shaker (170 rpm, 37 °C) for 2 h. The in vitro gastric digestion was followed by duodenal digestion. The pH was raised to 6.5 using NaOH and the following were added: 4 mmol/L sodium taurocholate, 4 mmol/L sodium glycodeoxycholate, 11.7 mmol/L CaCl_2_, 0.73 mmol/L Bis-Tris buffer (pH 6.5), 5.9 units/mL α-chymotrypsin, 104 units/mL trypsin, 3.2 μg/mL colipase, 54 units/mL pancreatic lipase and 25 units/mL alpha-amylase. The total volume of 340 mL was reached by the addition of saline (150 mmol/L NaCl, pH 6.5) and the final PC concentration was 2.1 mmol/L. The duodenal digestion was performed for 1 h in the shaking incubator (170 rpm, 37 °C). Then, samples were transferred to 1 kDa MWCO (molecular weight cut off) cellulose dialysis tubing (Spectra/Por^®^ 6, Spectrum Europe, Breda, Netherlands) and dialyzed overnight against NaCl (10 mmol/L) at 4 °C to remove low molecular mass digestion products. The dialysis fluid was changed and dialysis continued for an additional 2 h. Finally, apples were frozen at −20 °C and then freeze-dried until use. Inulin and cellulose (19.2 g each, equivalent with the dry content of 96 g of apples) were also digested and dialyzed using the same protocol.

### 2.6. Fecal Batch-Culture Fermentation and Samples Collection

The fermentation profile of the three commercial apples, the prebiotic inulin and the poorly fermented cellulose was determined using anaerobic, stirred, pH and temperature controlled fecal batch cultures. Glass water-jacketed vessels (300 mL) were sterilized and filled aseptically with 180 mL of pre-sterilized basal nutrient medium according to Sanchez-Patan et al. 2012 [22]. The pH was adjusted to 5.5–6.0 and kept between this range throughout the experiment with the automatic addition of NaOH or HCI (0.5 M), to mimic the conditions located in the proximal region of the human large intestine. The medium was then gassed overnight with oxygen free nitrogen to maintain anaerobic conditions. The following day and before the inoculation, each of the 5 vessels was dosed with 2 g of the appropriate substrate/treatment (inulin, cellulose, Renetta Canada, Golden Delicious or Pink Lady) for a final concentration of 1% (*w*/*v*). Fresh human fecal samples were collected in an anaerobic jar and were processed within 1 h. Fecal slurry was prepared by homogenizing the feces in pre-reduced phosphate buffered saline (PBS). The temperature was set to 37 °C using a circulating water-bath and the vessels were inoculated with 20 mL fecal slurry (10% *w*/*v* of fresh human feces) to a final concentration of 1% (*w*/*v*). Batch cultures were run under these controlled conditions for a period of 24 h, during which samples were collected at 4 time points (0, 5, 10 and 24 h) for FISH, SCFA, precursors polyphenols and polyphenol microbial metabolites. Pellets were stored at −80 °C for DNA extraction. Fermentations were conducted in triplicate using three healthy fecal donors.

### 2.7. DNA Extraction, Amplification and Sequencing

DNA was extracted from each sample (available for 0, 10 and 24 h time points) using the FastDNA Spin Kit for Feces (MP Biomedicals, UK). Nucleic acid purity was tested on NanoDropTM 8000 Spectrophotometer (Thermo Fisher Scientific). Total genomic DNA was then subjected to PCR amplification by targeting a ~460-bp fragment of the 16S rRNA variable region V3-V4 using the specific bacterial primer set 341F (5′ CCTACGGGNGGCWGCAG 3′) and 806R (5′ GACTACNVGGGTWTCTAATCC 3′) with overhang Illumina adapters. PCR amplification of each sample was carried out using 25 µL reactions with 1 µM of each primer, following the Illumina Metagenomic Sequencing Library Preparation Protocol for 16S Ribosomal RNA Gene Amplicons. The PCR products were checked on 1.5% agarose gel and cleaned from free primers and primer dimer using the Agencourt AMPure XP system (Beckman Coulter, Brea, CA, USA) following the manufacturer’s instructions. Subsequently dual indices and Illumina sequencing adapters Nextera XT Index Primer (Illumina) were attached by 7 cycles PCR (16S Metagenomic Sequencing Library Preparation, Illumina). The final libraries, after purification by the Agencourt AMPure XP system (Beckman), were analyzed on a Typestation 2200 platform (Agilent Technologies, Santa Clara, CA, USA) and quantified using the Quant-IT PicoGreen dsDNA assay kit (Thermo Fisher Scientific) by the Synergy2 microplate reader (Biotek). Finally, all the libraries were pooled in an equimolar way in a final amplicon library and analyzed on a Typestation 2200 platform (Agilent Technologies, Santa Clara, CA, USA). Barcoded library was sequenced on an Illumina^®^ MiSeq (PE300) platform (MiSeq Control Software 2.0.5 (Illumina, San Diego, CA, USA) and Real-Time Analysis software 1.16.18 (Illumina, San Diego, CA, USA)).

### 2.8. Sequence Data Analysis

Demultiplexed sequences were further processed using the Quantitative Insight Into Microbial Ecology (QIIME) open-source software package [23] using the following workflow: Forward and reverse Illumina reads (300 bp each) were joined using the fastq-join method [24], quality filtering was performed using 19 as the minimum Phred quality score and chimeric sequences were identified and removed using usearch 6.1. Then, sequences were assigned to operational taxonomic units (OTUs) using the QIIME implementation of UCLUST algorithm at 97% similarity threshold [25]. Representative sequences for each OTU were assigned to different bacterial taxonomic levels -phylum (p.), class (c.), order (o.), family (f.) and genus (g.)—by using Greengenes database release (May 2013).

The number of sequences collected that fulfilled quality control requirements (Phred quality score ≥20) yielded 1,647,935 (Sequence length mean ± SD, 450 ± 12). After removing chimeric sequences, a total of 1,621,799 reads remained, meaning that the used usearch61 algorithm reduced the dataset by approximately 1.6%. Using 97% as a homology cutoff value 4530 species-level OTUs were identified. For alpha and beta diversity tests all samples were subsampled to an equal number of reads (11,708 reads per sample which constitutes to 90% of the most indigent sample in the dataset). For further downstream analysis, the dataset was filtered to consider only those OTUs that were present in all samples at a relative abundance >0.005% (486 OTUs).

### 2.9. Enumeration of Bacterial Groups with Fluorescence In Situ Hybridization (FISH)

Changes in bacterial populations were determined using genus- and group-specific 16S rRNA gene-targeted oligonucleotide probes, labeled with Cy3 fluorescent dye, applying the FISH method [22]. The used oligonucleotide probes were: Bif164 specific for the *Bifidobacterium* spp. [26] and Fpra655 specific for the *Faecalibacterium prausnitzii* genus [27]. For total bacterial cell stain, the fixing of the samples onto the Teflon slides was performed as normal and the slides were incubated for 10 minutes in 50 mL of PBS with the addition of 50 μL of SYBR Green to a final concentration of 1:1000 [28].

### 2.10. Analysis of Short Chain Fatty Acids (SCFAs)

Analysis of SCFAs was performed using the method by Zhao et al. (2006) [29] with slight modifications. Briefly, 1 mL aliquots of 10% (*w*/*v*) fecal suspension in sterile 1 M PBS (pH 7.2) were dispensed into 1.5 mL tubes and centrifuged at 13,000× *g* for 5 min to pellet bacteria and other solids. Supernatants were then transferred into clean 1.5 mL tubes and frozen at 20 °C until required. On the day of the analysis samples were defrosted on ice and acidified to pH 2–3 by the addition of one volume of 6 M HCl to three volumes of sample. After 10 min incubation at room temperature, samples were centrifuged at 13,000× *g* for 5 min and filtered using a 0.2 µm polycarbonate syringe. One volume of 10 mM 2-ethylbutyirc acid was added to four volumes of sample as the internal standard. Calibration was done using standard solutions of acetic acid, propionic acid, i-butyric acid and n-butyric acid (Sigma-Aldrich, Schnelldorf, Germany) in acidified water (pH 2). SCFAs were determined by gas-liquid chromatography coupled with mass spectrometry on a Thermo Trace GC Ultra (Thermo Fisher Scientific, Austin, TX, USA) fitted with a FFAP column (Restek Stabilwax-DA; 30 m × 0.25 mm; 0.25 µm fth) and a flame-ionization detector. Peaks were integrated using Thermo Scientific Xcalibur data system (Thermo Fisher Scientific, Austin, TX, USA). All SCFAs showed a linear range between at least 0.5–20 mM with a coefficient of linearity *R*^2^ > 0.999. LOD and LOQ were below 0.5 mM.

### 2.11. Analysis of Precursor Polyphenols and Polyphenol Microbial Metabolites

The determination of precursor polyphenols was performed according to Vrhovsek et al. (2012) [19] whereas the polyphenol microbial metabolites according to Gasperotti et al. (2014) [20]. Briefly, a previously developed targeted metabolomic method was performed with an ultra-performance liquid chromatographic system coupled to tandem mass spectrometry system with electrospray ionization (UHPLC-ESI-MS/MS). Before injection, batch culture supernatants were defrosted, centrifuged (13,000 rpm, 5 min), filtered (0.22 μm) and trans-cinnamic acid-d5 (5 μg/mL) was added as the first internal standard. Then, samples were dried under nitrogen and reconstituted in methanol:water (1:1, *v*/*v*) containing rosmarinic acid (1 μg/mL) as the second internal standard. Samples were finally shaken for 10 min in an orbital shaker, centrifuged for 5 min at 16,000 rpm and injected (2 μL) into the UHPLC–MS/MS system. Data processing was performed using Waters MassLynx 4.1 (Waters, Milford, CT, USA) and TargetLynx software (Waters, Milford, CT, USA). Details of the liquid chromatography and mass spectrometry are described in Vrhovsek et al. (2012) [19] and Gasperotti et al. (2014) [20].

### 2.12. Statistical Analysis

For the sequencing data analysis, the QIIME pipeline version 1.9.1 [23] was used. Within community diversity (alpha diversity) was calculated using observed OTUs, Chao1 and Shannon indexes with 10 sampling repetitions at each sampling depth. Analysis of similarity (ANOSIM) and the ADONIS test were used to determine statistical differences between samples (beta diversity) following the QIIME compare_categories.py script and using weighted and unweighted phylogenetic UniFrac distance matrices. Principal Coordinate Analysis (PCoA) plots were generated using the QIIME beta diversity plots workflow. The biplot function was used to visualize samples and taxa in the PCoA space. For the rest of the data analysis the SPSS IBM version 21 (SPSS Inc., Chicago, IL, USA) was used. One-way ANOVA was used to determine differences between fermentation treatments (inulin, cellulose, Renetta Canada, Golden Delicious and Pink Lady) at the same time point (0, 5, 10 or 24 h), followed by the least significant difference (LSD) *post hoc* test. A repeated measures ANOVA was used to explore the differences within the same treatment/vessel (inulin, cellulose, Renetta Canada, Golden Delicious or Pink Lady) with all the time points (0, 5, 10 and 24 h) as within factor and with LSD as the *post hoc* test. In addition to these analyses, the *p* values were corrected using false discovery rate (FDR) to account for multiple testing at the lower bacterial taxonomical level (67 taxa). *p* ≤ 0.05 was considered statistically significant.

## 3. Results

### 3.1. Composition of Apples

The fiber and polyphenol content of the three fresh apples is shown in Table 1 (detailed nutrient composition analysis is presented in the Supplementary File, Table S1). Renetta Canada had the highest total polyphenol content (276 mg/100 g) followed by Golden Delicious (132 mg/100 g) and Pink Lady (94 mg/100 g). The total fiber content was similar among the apple varieties (Table 1).

### 3.2. Changes in Fecal Bacterial Alpha and Beta Diversity

**Figure 1:**
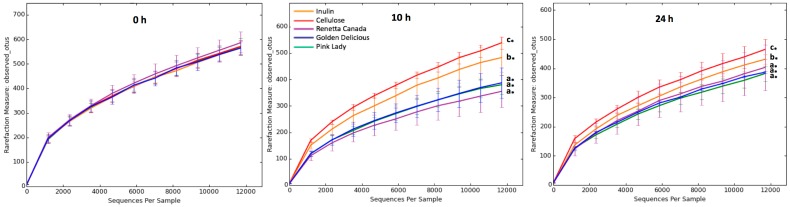
Alpha diversity (within-sample richness) rarefaction curves based on the observed number of Operational Taxonomic Units, OTUs (image), average Chao1 and Shannon indexes (±SEM) in 24-h in vitro batch culture fermentations inoculated with human feces (*n* = 3 healthy donors) and administrated with inulin, cellulose, Renetta Canada, Golden Delicious and Pink Lady as the substrates (treatments). Samples were analyzed at 0, 10 and 24 h. Ten sampling repetitions were calculated at an even sampling depth of 11708 sequences. Significant differences (*p* < 0.05) between treatments at the same time point are indicated with different letters. * Significant differences (*p* < 0.05) from the 0 h time point within the same treatment.

The diversity of gut microbiota within a community was measured with alpha diversity indices (within-sample richness), in particular the number of observed OTUs, the Chao1 estimator of species richness and the Shannon entropy and these are shown in Figure 1. At 0 h there were no differences between the treatments. At 10 h the observed OTUs, species richness (Chao1) as well as Shannon entropy were significantly lower with all the apple treatments compared to inulin or cellulose. At 24 h the same statistical differences as the 10 h time point were shown for the observed OTUs and species richness, with the exception of Shannon entropy, where Renetta Canada and inulin had lower values compared to the other apples or cellulose (*p* < 0.05). All alpha diversity indices decreased over time within every treatment throughout the fermentation (*p* < 0.05).

**Figure 2:**
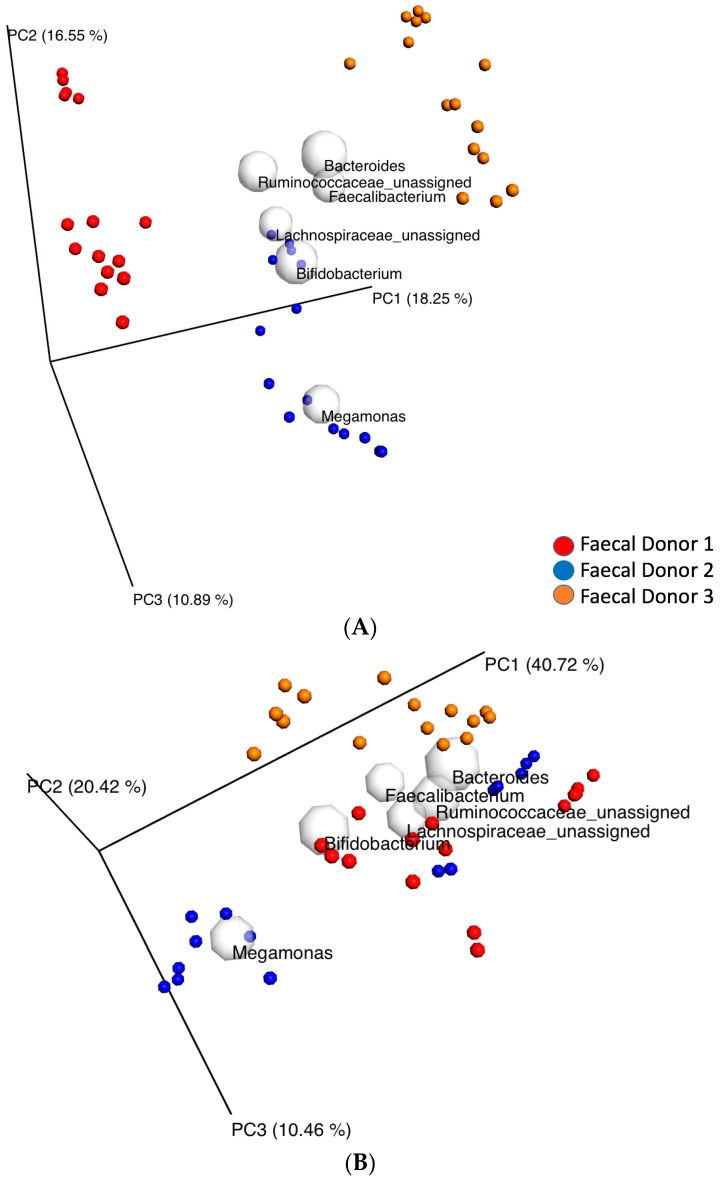
Principal coordinate analysis (PCoA) plots of 16S rRNA gene profiles based on (**A**) unweighted (qualitative) and (**B**) weighted (quantitative) phylogenetic UniFrac distance matrices calculated from a rarefied OTU table (11708 reads per sample) showing a clustering between donors (ANOSIM and ADONIS test, *p* = 0.01 and *p* = 0.001, respectively) for the whole data set (24-h in vitro batch culture fermentations inoculated with human feces (*n* = 3 healthy donors) and administrated with inulin, cellulose, Renetta Canada, Golden Delicious and Pink Lady as the substrates/treatments). Samples were analyzed at 0, 10 and 24 h. Each color represents a different donor. The gray spherical coordinates indicate taxonomic vectors of the 6 most prevalent taxa at the genus level. The size of each sphere is proportional to the mean relative abundance and approximates the causing variance throughout the plotted samples.

When the bacterial diversity between samples (for all the data set) was examined (beta diversity) a clustering was shown according to fecal donor (ANOSIM and ADONIS test, *p* = 0.01 and *p* = 0.001 (*R*^2^ = 34%), respectively) (Figure 2) and time point (ANOSIM and ADONIS test, *p* = 0.01 and *p* = 0.001 (*R*^2^ = 11%), respectively) (Figure S1), as demonstrated with principal coordinate analysis (PCoA) based on an unweighted (qualitative) phylogenetic UniFrac distance matrix. The clustering was less distinct but still significant according to donor (ANOSIM and ADONIS test, *p* = 0.01 and *p* = 0.001 (*R*^2^ = 29%), respectively) and time (ANOSIM and ADONIS test, *p* = 0.01 and *p* = 0.001 (*R*^2^ = 30%), respectively) when based on a weighted (quantitative) phylogenetic UniFrac distance matrix (Figure 2 and Figure S1, respectively). There was no significant effect of treatment on beta diversity for all the data set together (all time points and donors), ANOSIM test, *p* = 0.81 and *p* = 0.59 and ADONIS test, *p* = 0.95, *R*^2^ = 7% and *p* = 0.55, *R*^2^ = 8.5%, according to an unweighted and a weighted UniFrac distance matrix, respectively (Figure S2). The 6 core genera which influenced overall variance the most in the samples were *Bacteroides, Bifidobacterium, Megamonas, Ruminococcaceae* unassigned genus, *Lachnospiraceae* unassigned *genus and Faecalibacterium* (Figure 2, Figures S1 and S2).

### 3.3. Fecal Bacterial Relative Abundance at the Phylum Level

**Figure 3:**
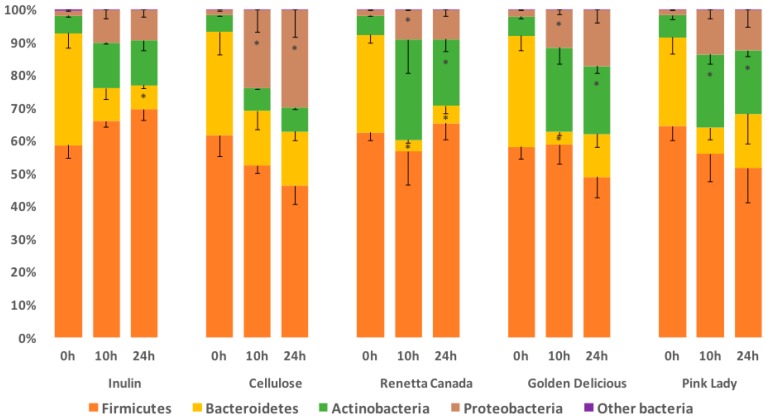
Changes in bacterial phyla (relative abundances (%)) throughout 24-h in vitro batch culture fermentations inoculated with human feces (*n* = 3 healthy donors) and administrated with inulin, cellulose, Renetta Canada, Golden Delicious and Pink Lady as the substrates (treatments). Samples were analyzed at 0, 10 and 24 h. Values are mean (%) with SEM (the negative error value is shown). Other bacteria represent Cyanobacteria, Lentisphaerae, Tenericutes and an unassigned phylum. * Significant differences from the 0 h time point within the same treatment (*p* < 0.05, FDR corrected).

The total sequence reads used in this study were classified into 7 phyla and one phylum was noted as unassigned. In particular, the bacterial communities, at time 0 h in all cultures, were dominated by bacteria belonging to Firmicutes (58–64%), Bacteroidetes (27–34%), Actinobacteria (5–7%) and Proteobacteria (1.5–2.0%) phylum, whereas a small percentage (0.1–0.3%) belonged to Cyanobacteria, Lentisphaerae, Tenericutes and an unassigned phylum (Figure 3). Treatment did not have any significant effect on the relative abundance of phylum level at time 0 h and 10 h. However, at time 24 h Actinobacteria relative abundance differed significantly among all five treatments (*p* = 0.017), where supplementation with all the apple varieties led to a higher abundance compared to cellulose (*p* < 0.05). Focusing on changes over time for each treatment separately (Figure 3), Firmicutes abundance remained unaffected, whereas Bacteroidetes significantly decreased over time with inulin (*p* = 0.012), Renetta Canada (*p* = 0.002) and Golden Delicious (*p* = 0.019). Actinobacteria proportion was significantly increased over time with all the apple varieties (Renetta Canada, *p* = 0.05, Golden Delicious, *p* = 0.011 and Pink Lady, *p* = 0.018). Finally, Proteobacteria abundance was also significantly increased with cellulose (*p* = 0.021), Renetta Canada (*p* = 0.012) and Golden Delicious (*p* = 0.02) (Figure 3).

### 3.4. Fecal Bacterial Relative Abundance at the Genus Level

At the lowest taxonomic level, 67 distinct bacterial taxa were detected. Of these, 46 were identified at the genus level, 15 at the family level, 5 at the order level and one was unassigned. At 0 h there were no differences between the treatments in bacterial taxa relative abundance. At 10 h, treatment had an effect on the abundance of g. *Oscillospira*, g. *Ruminococcus*, g. *Parabacteroides*, g. *Bilophila*, unassigned f. *Lachnospiraceae*, unassigned f. *Mogibacteriaceae* and unassigned and unclassified o. Clostridiales, which remained significant after the FDR correction for multiple testing for o. unassigned Clostridiales and f. *Mogibacteriaceae*, with cellulose administration showing higher proportions of these taxa compared to the other treatments (Table 2). Notably, *Bifidobacterium* g. abundance differed among all treatments at 10 h, with the highest proportion after Renetta Canada and Golden Delicious administration, however, this lost significance with FDR correction (Table 2). Significant differences, before correction, between treatments were also observed at 24 h on the relative abundance of g. *Faecalibacterium*, g. *Butyricimonas*, g. *Bifidobacterium* and unassigned o. Clostridiales. Additional details on the relative abundance of bacterial taxa at 10 and 24 h for the different treatments are shown in Table 2. Focusing on changes over time for each treatment separately, there were significant changes in the relative abundance of specific taxa however, these were not always significant after correction and presented as supplementary information (Table S2).

### 3.5. Changes in Selected Fecal Bacterial Populations Measured with FISH

**Figure 4:**
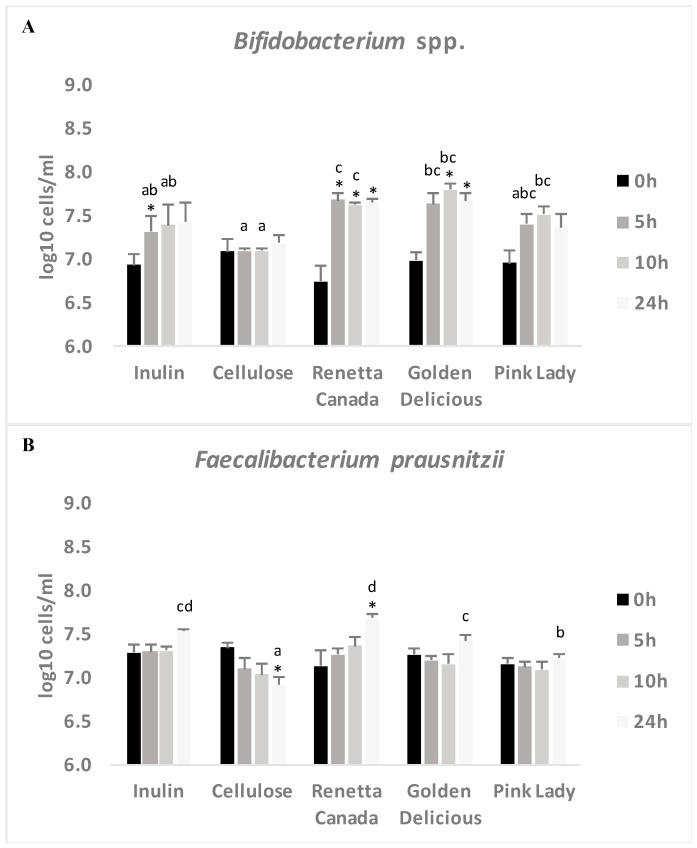

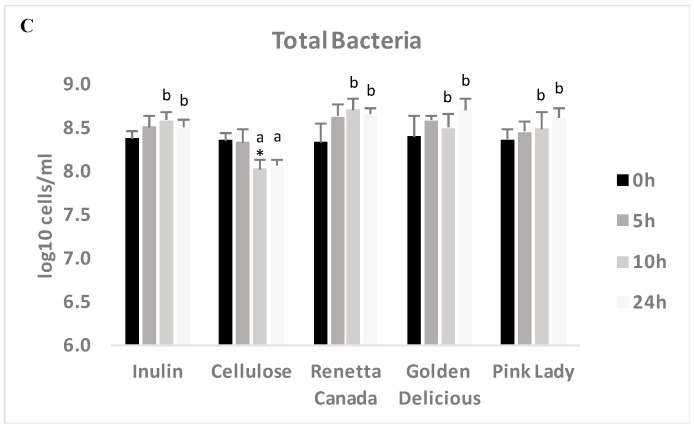
Changes in bacterial populations ((**A**) *Bifidobacterium* spp.; (**B**) *Faecalibacterium prausnitzii* and (**C**) Total Bacteria) throughout 24-h in vitro batch culture fermentations inoculated with human feces (*n* = 3 healthy donors) and administrated with inulin, cellulose, Renetta Canada, Golden Delicious and Pink Lady as the substrates (treatments). Samples were collected at 0, 5, 10 and 24 h. Results are expressed as log_10_ cells/mL of batch culture medium and values are mean ± SEM of the three fermentations. Significant differences (*p* < 0.05) between treatments at the same time point are indicated with different letters. * Significant differences (*p* < 0.05) from the 0 h time point within the same treatment.

Changes in *Bifidobacterium* spp., *Faecalibacterium prausnitzii* and total bacteria were also assessed by FISH (Figure 4). At 0 h there were no significant changes between the treatments. At 5 h bifidobacteria numbers increased significantly with Renetta Canada compared to cellulose (*p* = 0.004) and inulin (*p* = 0.047); bifidobacteria also increased with Golden Delicious as the treatment compared to cellulose (*p* = 0.007). At 10 h bifidobacteria and total bacteria increased significantly with all the apple varieties compared to cellulose (*p* < 0.05); with total bacteria also increasing with inulin compared to cellulose (*p* = 0.009). Bifidobacteria also increased at 10 h with Renetta Canada compared to inulin (*p* = 0.036). At 24 h *Faecalibacterium prausnitzii* increased significantly with Renetta Canada compared to the other apples (*p* < 0.05). All apple varieties and inulin increased *Faecalibacterium prausnitziii* compared to cellulose (*p* < 0.05). Inulin and Golden Delicious also had higher *Faecalibacterium prausnitziii* numbers at 24 h compared to Pink Lady (*p* = 0.004 and *p* = 0.032 respectively) (Figure 4). Finally, at 24 h total bacteria increased significantly with all the apple varieties and inulin compared to cellulose (*p* < 0.05).

Following changes over time for the same treatment, a significant increase in bifidobacteria population, from 0 h, was observed for Renetta Canada (compared to 5, 10 and 24 h (*p* < 0.05)) and Golden Delicious (compared to 10 and 24 h (*p* < 0.05)). Furthermore, inulin also increased *Bifidobacterium* spp. at 5 h (*p* = 0.044) compared to the 0 h value, but to a lesser extent compared to Renetta Canada and Golden Delicious. *Faecalibacterium prausnitzii* population was significantly higher after 24 h only for Renetta Canada compared to 0 h (*p* = 0.049), while it decreased significantly after the administration of cellulose (at 24 h compared to 0 h, *p* = 0.02). Apart from the cellulose treatment (significant decrease at 10 h, *p* = 0.028) there were no significant changes over time in total bacteria population with any of the other treatments (Figure 4).

### 3.6. SCFAs Production

Changes in SCFAs concentrations over time with the different treatments are shown in Table 3. All apples varieties significantly increased the concentration of acetic, propionic and total SCFAs (*p* < 0.05), but only Renetta Canada increased butyric acid among the apples (*p* < 0.05). Inulin significantly increased the concentrations of acetic, butyric and total SCFAs (*p* < 0.05) but these remained lower compared to the apple varieties. Cellulose increased butyric acid and total SCFAs but to a much lesser extent compared to inulin and the apple varieties (*p* < 0.05). There were no significant changes between the treatments at the same time point (0, 5, 10 or 24 h).

### 3.7. Changes in Precursor Polyphenols

A list of the precursor polyphenols and polyphenol microbial metabolites together with their multiple reaction monitoring (MRM) conditions are presented in Table S3. Changes in the concentration of precursor polyphenols, during the fecal fermentation, are shown in Table S4. Proanthocyanidin, kaempferol-3-rutinoside, rutin, isorhamentin-3-glucoside and cyanidin 3-galactoside were measured only in fresh apples whereas procyanidin A2, quercetin, kaempferol, isorhamnetin, laricitrin, phloretin, luteolin and ellagic acid were measured only in batch cultures.

Changes between the three apple varieties were observed at 0 h. In particular, Renetta Canada treatment resulted in significant higher concentrations of (+)-catechin, (−)-epicatechin, procyanidin A2, procyanidin B1, phloretin, phlorizin and vanillin compared to Golden Delicious and Pink Lady (Table S4). On the other hand, treatment with Golden Delicious resulted in higher (*p* < 0.05) levels of quercetin-3-glc compared to Pink Lady and Renetta Canada and higher quercetin-3-rha compared to Renetta Canada (Table S4). There were no significant changes in the concentration of precursor polyphenols at 5, 10 or 24 h.

Changes in the concentration of precursor polyphenols were observed over time throughout the fecal fermentation of the three apple varieties. In particular, significant reductions throughout the fermentation were detected for (+)-catechin (Renetta Canada), (−)-epicatechin (Renetta Canada and Golden Delicious), procyanidin A2 (Renetta Canada, Golden Delicious and Pink Lady), neochlorogenic acid (Golden Delicious), cryptochlorogenic acid (Golden Delicious), quercetin-3-glc (Renetta Canada, Golden Delicious and Pink Lady), quercetin-3-rha (Renetta Canada, Golden Delicious and Pink Lady), kaempferol (Renetta Canada), isorhamnetin (Renetta Canada), phlorizin (Renetta Canada and Golden Delicious) and vanillin (Renetta Canada and Golden Delicious) (Table S4).

### 3.8. Formation of Polyphenol Microbial Metabolites

**Figure 5:**
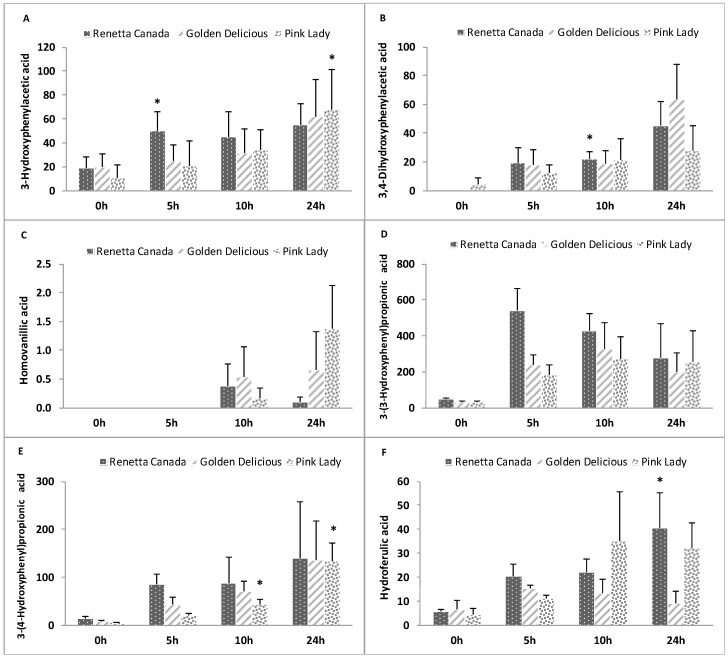
Changes in phenylacetic ((**A**) 3-Hydroxyphenylacetic acid; (**B**) 3,4-Dihydroxyphenylacetic acid and (**C**) Homovanillic acid) and phenylpropionic acid ((**D**) 3-(3-Hydroxyphenyl)propionic acid; (**E**) 3-(4-Hydroxyphenyl)propionic acid and (**F**) Hydroferulic acid) derivatives throughout 24-h in vitro batch culture fermentations inoculated with human feces (*n* = 3 healthy donors) and administrated with Renetta Canada, Golden Delicious and Pink Lady as the substrates (treatments). Samples were collected at 0, 5, 10 and 24 h. Results are expressed as ng/mL of batch culture medium and values are mean ± SEM of the three fermentations. * Significant differences (*p* < 0.05) from the 0 h time point within the same treatment.

**Figure 6:**
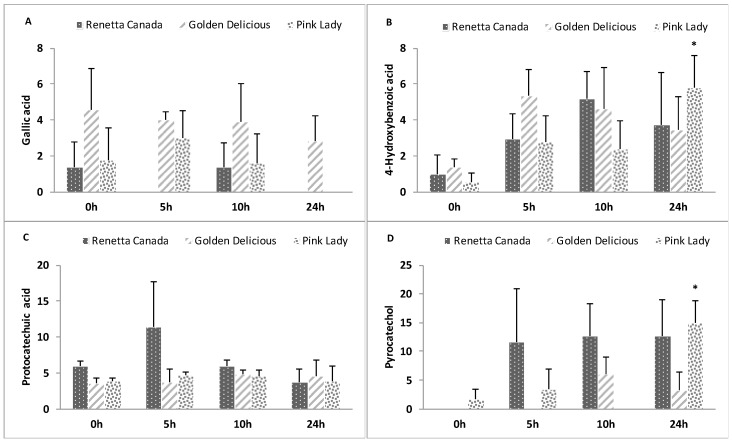
Changes in benzoic acid derivatives ((**A**) Gallic acid; (**B**) 4-Hydroxybenzoic acid; (**C**) Protocatechuic acid and (**D**) Pyrocatechol) throughout 24-h in vitro batch culture fermentations inoculated with human feces (*n* = 3 healthy donors) and administrated with Renetta Canada, Golden Delicious and Pink Lady as the substrates (treatments). Samples were collected at 0, 5, 10 and 24 h. Results are expressed as ng/mL of batch culture medium and values are mean ± SEM of the three fermentations. * Significant differences (*p* < 0.05) from the 0 h time point within the same treatment.

The formation of polyphenol microbial metabolites throughout the fecal fermentation of the three apple varieties is shown in Figure 5 and Figure 6. Significant increases were observed over time for 3-hydroxyphenylacetic acid (Renetta Canada and Pink Lady, *p* = 0.034 and *p* = 0.043, respectively), 3,4-dihydroxyphenylacetic acid (Renetta Canada, *p* = 0.05), 3-(4-hydroxyphenyl)propionic acid (Pink Lady, *p* = 0.009), hydroferulic acid (Renetta Canada, *p* = 0.046), 4-hydroxybenzoic acid (Pink Lady, *p* = 0.017) and pyrocatechol (Pink Lady, *p* = 0.049). In contrast, significant decreases throughout the fermentation were shown for caffeic acid (Renetta Canada and Golden Delicious, *p* = 0.000 and *p* = 0.001, respectively), p-coumaric acid (Renetta Canada and Golden Delicious, *p* = 0.001 and *p* = 0.001, respectively), trans-ferulic (Renetta Canada and Golden Delicious, *p* = 0.002 and *p* = 0.003, respectively) and trans-isoferulic (Renetta Canada, *p* = 0.001), as these metabolites can also appear as precursor polyphenols in apples (Figure S3). There were no significant changes in the concentration of the polyphenol microbial metabolites between the three apple varieties when each time point (0, 5, 10 or 24 h) was explored separately, with the exception of caffeic acid and p-coumaric acid (significantly higher concentration with Renetta Canada fermentation compared to Golden Delicious and Pink Lady at 0 h) and t-ferulic acid (significantly higher concentration with Renetta Canada compared to Pink Lady at 0 h).

## 4. Discussion

The present in vitro study showed that whole apples can effectively modify both the human fecal microbiota composition and metabolic output. Effects on the bacterial community were observed at phylum and genus/species level. Actinobacteria relative abundance increased with all the tested apple varieties (Renetta Canada, Golden Delicious and Pink Lady). Increases in Actinobacteria have been observed in humans after intake of pectin [30], resistant starch [31] and pomegranate extract [32] and in rats fed with wild blueberries [33]. This increase can be explained by *Bifidobacterium* spp. growth, an important member of the Actinobacteria phylum. Although this did not remain significant with Illumina sequencing after multiple testing correction in the current study, the FISH results showed that *Bifidobacterium* spp. population increased significantly after the administration with Renetta Canada and Golden Delicious varieties. Notably, *Bifidobacterium* is considered a beneficial member of the gut microbiota by inhibiting the growth of pathogens, synthesizing certain vitamins (e.g., folate) and reducing serum cholesterol [2]. This observation is consistent with previous studies showing a bifidogenic effect with extraction juices from apple pomace in rats [12] and with the administration of two apples daily for two weeks in eight human subjects [17]. In contrast, Masumoto et al. (2016) [15], using a high throughput metagenomics technique, have reported decreased relative abundance of *Bifidobacterium* in diet-induced obese mice after the administration of apple PAs [15]. In our study, inulin, a known prebiotic, increased bifidobacteria to a lesser extent than apples. Inulin structure can affect its utilization by gut bacteria and many isolated bifidobacteria cannot utilize long-chain inulin [8] but they can grow on short-chain length structures (i.e., fructo-oligosaccharides) [34]. The inulin in the current study was a commercial isolate from dahlia tubers and details of its structure were not available.

Within the Firmicutes phylum, *Faecalibacterium prausnitzii* population (measured with the quantitative FISH) increased with Renetta Canada administration. *F. prausnitzii* is a key butyrate-producer, with anti-inflammatory properties, that may offer potential health benefits, especially in patients with inflammatory bowel disease (IBD) [35,36]. Renetta Canada increased butyrate, a major energy source for the colonocytes, which is particularly beneficial to the gut mucosa [37]. In support of our results *F. prausnitzii* strains have been shown to utilize apple pectin for growth [8,38] and increase butyrate concentration [13,39]. *F. prausnitzii* levels were unaffected by Golden Delicious and Pink Lady, and although the concentration of pectin was not determined, Renetta Canada had 19% and 44% higher soluble fiber content compared with Golden Delicious and Pink Lady, respectively. These data suggest that at least for *F. prausnitzii,* pectin may have played a major role.

Bacteroidetes relative abundance decreased with inulin, Renetta Canada and Golden Delicious. *Bacteroides* is considered a dominant bacterial group in the large intestine and the main Bacteroidetes member, along with the *Prevotella*. Licht et al. (2010) reported that both whole apples and isolated pectin decreased *Bacteroides* spp. in rats compared to a control diet [13]. Moreover, *Bacteroides* has been shown to decrease after the administration of other polyphenol sources, such as red wine [40] and cocoa [41] in rats, as well as with grape [42] and date extracts [43] in in vitro gut models inoculated with human feces. By contrast, *Bacteroides* species have been shown to increase with apple pomace juice extracts [11] and PAs from *Acacia angustissima* [44] in rats, as well as with red wine in humans [45].

The proportion of Proteobacteria increased after the administration of Renetta Canada and Golden Delicious but to a lesser extent compared to cellulose. However, an increase in *Enterobacteriaceae* family, a major member of Proteobacteria, was not observed. *Enterobacteriaceae*, includes numerous pathogenic bacteria genera, such as *Escherichia*, *Salmonella* and *Yersinia* and has been shown to increase in IBD patients. Increased Proteobacteria with inulin [8], resistant starch [46] and de-alcoholized wine [45] has also been reported elsewhere.

Interestingly, the alpha diversity of gut microbiota, at the OTU level, was lower with the apple treatments compared to inulin or cellulose. This may indicate the selective nature of the apple fermentations towards particular species. The beta diversify analysis showed a partitioning by donor and time, but not with treatment, which indicates that each individual possesses a specific starting population of gut bacteria, a finding consistent with the previously described inter-individual variation in the intestinal microbiota [47,48]. However, despite the variability between donors, there were still treatment-associated changes in gut microbiota composition at phylum and at genus/species level.

In the present study, the conditions of the proximal colon were simulated by creating an environment moderately acidic (pH 5.5–6.0) compared to a more neutral pH in the transverse and distal colon. The majority of the unabsorbed dietary carbohydrates are fermented in the proximal section producing SCFAs, leading to this reduced colonic pH, whereas in the distal section carbohydrate fermentation is generally assumed to be low. The pH affects bacterial growth and SCFA production, especially among bacteria that utilize the same polysaccharides [8,49]. For example, suppression in *Bacteroides* spp. growth was observed at pH values below six [8]. On the other hand, *F. prausnitzii* is more low-pH tolerant [8]. Moreover, a lower pH tends to favor butyrate production [49]. However, in our study, a significant increase in butyrate levels and *F. prausnitzii* population was only shown by Renetta Canada, indicating a treatment effect rather than a pH effect.

Pectin, the main soluble fiber found in apples, is extensively fermented by the gut microbiota to SCFAs, which are an important energy source for colonic health as well as for other tissues and organs [2]. Apart from the aforementioned butyrate increase by Renetta Canada, all apples significantly increased propionate and mainly acetate. Increased SCFAs have been shown with apple pomace juices [11,12]. Acetate serves as an energy source for the liver and peripheral tissues, but is also involved in the metabolic pathways of lipogenesis [50]. Pectin is known to produce relatively large amounts of acetate [51], which can also be utilized by butyrate producers such as *F. prausnitzii* as part of the cross feeding between bacteria [52]. A cross-feeding between *Bifidobacterium* strains and *F. prausnitzii* has been suggested, enhancing butyrate production [53]. Propionate on the other hand, may help to reduce hepatic cholesterol synthesis [54].

The effects of polyphenols on health depend on their bioavailability. Flavanol monomers (i.e., catechin and epicatechin) are readily absorbed in the small intestine, while high molecular weight polyphenols, such as the polymeric PAs, reach the colon almost intact, where they are transformed by the gut bacteria into a complex mixture of simple phenolic acids [55]. In the present study, the degradation of precursor apple polyphenols started as early as 5 h of fermentation and was complete throughout the 24 h for most of the polyphenolic compounds. Renetta Canada fermentation resulted in higher degradation of precursor polyphenols due to their initial high concentration.

The formation of polyphenol microbial products represent potential beneficial bioactive metabolites, not only locally in the gut but also systematically after their absorption in the colon and their appearance in the blood circulation. Renetta Canada was associated with the production of 3,4-dihydroxyphenylacetic acid and hydroferulic acid, which both have shown to possess anti-inflammatory properties [56]. It has been proposed that 3,4-dihydroxyphenylacetic acid can arise from the microbial catabolism of dimeric PAs [57]. Moreover, microbial metabolites of chlorogenic acids such as dihydroferulic acid showed a high antioxidant activity in vitro [58]. These results are in line with the higher concentration of PAs and chlorogenic acid in Renetta Canada apples. Pink Lady was associated with the formation of 3-(4-hydroxyphenyl)propionic acid and benzoic acid derivatives, in particular 4-hydroxybenzoic acid and pyrocatechol. Benzoic acids such as 4-hydroxybenzoic acid are considered to arise from beta oxidation of phenylpropionic acid derivatives and higher levels have been found after the in vitro fermentation of grape seed flavanols [59]. Pyrocatechol may arise from the dehydroxylation of gallic acid [60], which has been identified as a microbial metabolite and a native compound [61]. In our study, gallic acid concentration remained unaffected throughout the fermentation. Finally, both Renetta Canada and Pink Lady apples increased 3-hydroxyphenylacetic acid concentration. In vitro studies with human fecal inoculum are in line with the identified phenolic acids. In particular, PA catabolism has been associated with the production of 3-hydroxyphenylpropionic acid, 3-phenylpropionic acid, 4-hydroxyphenylpropionic acid and 4-hydroxyphenylacetic acid [62] as the main metabolites, whereas apples and apple components including isolated PAs formed 3-(3,4-dihydroxyphenyl)propionic acid, 3-(3-hydroxyphenyl)propionic acid, 3-phenylpropionic acid, benzoic acid, 2-(3,4-dihydroxyphenyl)acetic acid and 2-(3-hydroxyphenyl)acetic acid [10]. Furthermore, in human subjects, chocolate intake, a rich source of flavanols increased the urinary excretion of 3-hydroxyphenylpropionic acid, ferulic acid, 3,4-dihydroxyphenylacetic acid, 3-hydroxyphenylacetic acid, vanillic acid and 3-hydroxybenzoic acid [63].

In this study, we demonstrated that whole apples could modify the gut microbiota composition and affect the extent of degradation of soluble fiber and polyphenols through the production of SCFAs and phenolic acids, with Renetta Canada variety showing the most beneficial effects. In vitro batch culture models are a quick, simple and cost effective method of mimicking changes in gut microbiota numbers and metabolism [64], although they lack key metabolic functions, such as host immunological interactions, intestinal absorption and physiological components, such as epithelial mucosa, that exist in the human colon. The sample size (*n* = 3) is consistent with similar studies investigating polyphenols extracts [22,59], prebiotics [65,66] and fruits [43,67], with observed changes consistent with outcomes of human intervention studies [68]. Batch culture vessels contain a basal medium with limited carbohydrate and protein sources, therefore changes in microbiota composition and fermentation metabolites is known to be due to the added substrate, the apple varieties added to the vessels, even with different starting bacterial populations. Finally, although, donors were of similar age, with no gastrointestinal disorders, other characteristics that may affect gut microbiota composition such as diet, exercise and stress levels were not recorded and may have influenced the observed results, and it is recommended that these are provided in future studies.

## 5. Conclusions

In conclusion, whole apples beneficially modulate the gut microbiota composition and metabolic output in vitro. Renetta Canada variety in particular may have positive consequences for human health by increasing bifidobacteria, *Faecalibacterium prausnitzii* population and producing SCFAs and polyphenol microbial metabolites. It is recommended that the findings of this in vitro study should be confirmed in human intervention trials.

## Figures and Tables

**Table:** 

Treatment	Chao1 (0 h)	Shannon (0 h)		Chao1 (10 h)	Shannon (10 h)		Chao1 (24 h)	Shannon (24 h)
Inulin	1076 ± 43	6.36 ± 0.36		931 ± 91 ^b,^*	5.32 ± 0.53 ^b,^*		811 ± 37 ^b,^*	4.88 ± 0.32 ^a,^*
Cellulose	1091 ± 26	6.39 ± 0.43		1030 ± 66 ^c,^*	5.99 ± 0.27 ^c,^*		849 ± 89 ^c,^*	5.72 ± 0.29 ^c,^*
Renetta Canada	1124 ± 84	6.49 ± 0.28		673 ± 113 ^a,^*	4.28 ± 0.71 ^a,^*		774 ± 135 ^a,^*	4.78 ± 0.63 ^a,^*
Golden Delicious	1064 ± 46	6.35 ± 0.41		724 ± 125 ^a,^*	4.57 ± 0.64 ^a,^*		737 ± 31 ^a,^*	5.20 ± 0.07 ^b,^*
Pink Lady	1115 ± 67	6.47 ± 0.27		748 ± 69 ^a,^*	4.63 ± 0.66 ^a,^*		769 ± 48 ^a,^*	5.14 ± 0.47 ^b,^*

**Table 1:** Composition analysis of Renetta Canada, Golden Delicious and Pink Lady *.

Components	Renetta Canada	Golden Delicious	Pink Lady
Total dietary fiber (AOAC) (g/100 g)	2.6	2.4	2.4
Soluble fiber (AOAC) (g/100 g)	1.6	1.3	0.9
Insoluble fiber (AOAC) (g/100 g)	1.0	1.1	1.5
*Polyphenols (mg/100 g)*			
*Flavanols*			
(+)—Catechin	1.07	0.16	0.17
(−)—Epicatechin	10.9	2.8	2.8
Procyanidin B1	6.6	0.95	0.78
Procyanidin B2 + B4 (as B2)	18.3	6.1	4.8
Proanthocyanidin (as cyanidin)	169.2	91.5	62.1
*Hydroxycinnamates*			
Chlorogenic acid	61	18.7	17.5
Neochlorogenic acid	0.04	0.04	0.01
Cryptochlorogenic acid	0.98	0.67	0.15
*Flavonols*			
Quercetin-3-glucoside	0.99	6.9	3.1
Quercetin-3-rhamnoside	0.65	2.7	1.7
Rutin	0.09	0.44	0.19
Kaempferol-3-rutinoside	0.002	0.011	0.002
Isorhamnetin-3-glucoside	0.001	0.002	0.001
*Dihydrochalcones*			
Phlorizin	5.9	1.5	0.8
*Anthocyanins*			
Cyanidin 3-galactoside	0.006	0.017	0.027
*Benzoic Acid Derivatives*			
Vanillin	0.008	0.006	0.003
Vanillic acid	0.001	0.003	0.002

* For each apple variety a mixture of three fresh whole apples was analyzed.

**Table 2:** Changes in bacterial taxa relative abundance (%) at 10 h and 24 h of in vitro batch culture fermentations inoculated with human feces (*n* = 3 healthy donors) and administrated with inulin, cellulose, Renetta Canada, Golden Delicious and Pink Lady as the substrates (treatments).

Phylum	Class	Order	Family	Genus	Time Point	Inulin (%)	Cellulose (%)	Renetta Canada (%)	Golden Delicious (%)	Pink Lady (%)	*p* *	*p* ^#^ (FDR-Corrected)
Firmicutes	Clostridia	Clostridiales	*Lachnospiraceae*	unassigned	10 h	4.3 ± 1.3 ^a^	9 ± 0.9 ^b^	2.6 ± 0.7 ^a^	5 ± 2 ^a,b^	2.9 ± 1.2 ^a^	0.040	0.336
Firmicutes	Clostridia	Clostridiales	[*Mogibacteriaceae*]	unassigned	10 h	0.15 ± 0.01 ^b^	0.14 ± 0.01 ^b^	0.08 ± 0.02 ^a^	0.05 ± 0.01 ^a^	0.06 ± 0.01 ^a^	0.001	0.040
Firmicutes	Clostridia	Clostridiales	*Ruminococcaceae*	*Oscillospira*	10 h	0.66 ± 0.1 ^b^	1 ± 0.1 ^c^	0.32 ± 0.03 ^a^	0.3 ± 0.1 ^a^	0.23 ± 0.07 ^a^	0.001	0.052
Firmicutes	Clostridia	Clostridiales	*Ruminococcaceae*	*Ruminococcus*	10 h	1.4 ± 0.3 ^a,b^	1.8 ± 0.5 ^b^	0.63 ± 0.43 ^a^	0.41 ± 0.18 ^a^	0.45 ± 0.17 ^a^	0.050	0.369
Firmicutes	Clostridia	Clostridiales	unassigned	unassigned	10 h	1.8 ± 0.1 ^b^	2.8 ± 0.3 ^c^	0.82 ± 0.24 ^a^	0.76 ± 0.16 ^a^	0.81 ± 0.12 ^a^	0.000	0.009
Firmicutes	Clostridia	Clostridiales	unclassified	unclassified	10 h	0.68 ± 0.12 ^b^	1.4 ± 0.23 ^c^	0.47 ± 0.1 ^a,b^	0.33 ± 0.03 ^a,b^	0.59 ± 0.27 ^a,b^	0.013	0.215
Bacteroidetes	Bacteroidia	Bacteroidales	*Porphyromonadaceae*	*Parabacteroides*	10 h	0.72 ± 0.2 ^b^	1.9 ± 0.6 ^c^	0.4 ± 0.1 ^a,b^	0.37 ± 0.04 ^a,b^	0.56 ± 0.1 ^a,b^	0.022	0.264
Actinobacteria	Actinobacteria	Bifidobacteriales	*Bifidobacteriaceae*	*Bifidobacterium*	10 h	4.7 ± 2.4 ^a^	2.8 ± 0.2 ^a^	24.3 ± 8.7 ^b^	19.4 ± 4.2 ^b^	16.2 ± 2.1 ^a,b^	0.028	0.273
Proteobacteria	Deltaproteobacteria	Desulfovibrionales	*Desulfovibrionaceae*	*Bilophila*	10 h	0.33 ± 0.03 ^a^	1.7 ± 0.6 ^b^	0.17 ± 0.01 ^a^	0.24 ± 0.08 ^a^	0.26 ± 0.05 ^a^	0.024	0.264
Firmicutes	Clostridia	Clostridiales	*Ruminococcaceae*	*Faecalibacterium*	24 h	17 ± 3.7 ^c^	4.6 ± 2.2 ^a^	16 ± 5.5 ^b,c^	5.9 ± 1.9 ^a,b^	5.4 ± 1.4 ^a^	0.049	0.950
Firmicutes	Clostridia	Clostridiales	unassigned	unassigned	24 h	1.2 ± 0.3 ^a^	2.5 ± 0.6 ^b^	0.36 ± 0.05 ^a^	0.75 ± 0.19 ^a^	0.86 ± 0.19 ^a^	0.007	0.482
Bacteroidetes	Bacteroidia	Bacteroidales	[*Odoribacteraceae*]	*Butyricimonas*	24 h	0.04 ± 0.01 ^a,b^	0.09 ± 0.03 ^b^	0.03 ± 0.01 ^a^	0.02 ± 0.01 ^a^	0.02 ± 0.01 ^a^	0.044	0.950
Actinobacteria	Actinobacteria	Bifidobacteriales	*Bifidobacteriaceae*	*Bifidobacterium*	24 h	6.3 ± 4.6 ^a,b^	2.6 ± 0.2 ^a^	14.9 ± 2.5 ^b^	15.2 ± 2.6 ^b^	10.4 ± 3 ^a,b^	0.050	0.950

* ANOVA analysis to verify whether the relative abundance of a given taxa is different between the treatments within the same time point. ^#^ The *p* value after correction for multiple tests (67 taxa) with the false discovery rate (FDR) method. Different letters (a, b, c) indicate significant differences (*p* < 0.05) between treatments at the same time point. Brackets indicate suggested but not verified names. Values are mean ± SEM.

**Table 3:** SCFA concentrations (mmol/L) throughout 24-h in vitro batch culture fermentations inoculated with human feces (*n* = 3 healthy donors) and administrated with inulin, cellulose, Renetta Canada, Golden Delicious and Pink Lady as the substrates (treatments).

Substrate	Time (h)	Acetic Acid (mmol/L)	*p* ^a^	Propionic Acid (mmol/L)	*p* ^a^	Butyric Acid (mmol/L)	*p* ^a^	Total SCFAs (mmol/L)	*p* ^a^
Inulin	0	2.2 ± 0.1	0.050	0.8 ± 0.1	0.185	1 ± 0.3	0.013	4.1 ± 0.6	0.049
	5	9.5 ± 5.4		2.8 ± 1.4		4.4 ± 2.8		16.8 ± 9.6	
	10	11 ± 2.1		2.9 ± 0.8		5.5 ± 1 *		19.3 ± 3.3 *	
	24	17.3 ± 2.1 *		6.9 ± 2.6		11 ± 0.5 *		35.4 ± 4.6 *	
Cellulose	0	4.8 ± 2.9	0.062	1.4 ± 0.8	0.062	1.2 ± 0.6	0.041	7.5 ± 4.2	0.022
	5	5 ± 1.3		1.9 ± 0.5		2.3 ± 0.2 *		9.2 ± 2.3	
	10	13.5 ± 5.2		3.3 ± 1		2.8 ± 0.6		19.5 ± 5.6 *	
	24	13.1 ± 1.1		3.4 ± 0.1		3.5 ± 1		20 ± 1.5	
Renetta Canada	0	1 ± 0.1	0.034	0.5 ± 0.1	0.020	0.5 ± 0.1	0.025	3 ± 0.3	0.044
	5	11.5 ± 2.7		4.6 ± 2.6		2 ± 0.5		18.1 ± 3.9	
	10	19 ± 2.2 *		5.6 ± 2.1		3.7 ± 0.6 *^,#^		28.4 ± 1.8 *	
	24	28.1 ± 6 *^,#^		8.8 ± 2.6 ^#,^^		16.9 ± 6.1		53.7 ± 11.4 *	
Golden Delicious	0	2 ± 0.4	0.034	0.6 ± 0.1	0.016	0.5 ± 0.2	0.057	3.1 ± 0.7	0.008
	5	13.9 ± 5.8		4.2 ± 1.6		1.9 ± 0.7		20 ± 6.7	
	10	15.3 ± 3 *		4.7 ± 1.9		4.9 ± 1.6		25 ± 3.1 *	
	24	23.7 ± 2.8 *		7.8 ± 1.9 ^#,^^		13.3 ± 5.1		44.8 ± 7.2 *^,#^	
Pink Lady	0	1.8 ± 0.3	0.049	0.5 ± 0.1	0.020	0.4 ± 0.1	0.044	2.7 ± 0.5	0.040
	5	8.3 ± 2.3		3.2 ± 1.5		1.1 ± 0.2		12.6 ± 2.9	
	10	22.8 ± 8		6 ± 2.2		4 ± 1.2		32.8 ± 9.7	
	24	26.2 ± 3.8 *^,#^		8.8 ± 2 ^#,^^		13 ± 2.8		48 ± 7.9 *^,^^#,^^	

Results are expressed as mmol/L of batch culture medium and values are mean ± SEM of the three fermentations. ^a^ Difference over time within the same treatment (ANOVA). * Significant different from 0 h time point *p* < 0.05, # Significant different from 5 h time point, *p* < 0.05, ^ Significant different from 10 h time point, *p* < 0.05.

## Supplementary Materials

The following are available online at www.mdpi.com/2072-6643/9/5/533/s1, Figure S1: Principal coordinate analysis (PCoA) plots of 16S rRNA gene profiles based on (A) unweighted (qualitative) and (B) weighted (quantitative) phylogenetic UniFrac distance matrices, colored by time, Figure S2: Principal coordinate analysis (PCoA) plots of 16SrRNA gene profiles based on (A) unweighted (qualitative) and (B) weighted (quantitative) phylogenetic UniFrac distance matrices, colored by treatment, Figure S3: Changes in cinnamic acid derivatives, Table S1: Composition analysis of Renetta Canada, Golden Delicious and Pink Lady, Table S2: Changes in bacterial taxa throughout 24-h in vitro batch culture fermentations inoculated with human feces (*n* = 3 healthy donors) and administrated with inulin, cellulose, Renetta Canada, Golden Delicious and Pink Lady as the substrates, Table S3: Multiple Reaction Monitoring (MRM) conditions of precursor polyphenols and polyphenol microbial metabolites, Table S4: Changes in precursor polyphenols throughout 24-h in vitro batch culture fermentations inoculated with human feces (*n* = 3 healthy donors) and administrated with inulin, cellulose, Renetta Canada, Golden Delicious and Pink Lady as the substrates.

## References

[1] Wang X., Ouyang Y.Y., Liu J., Zhu M.M., Zhao G., Bao W., Hu F.B. (2014). Fruit and vegetable consumption and mortality from all causes, cardiovascular disease, and cancer: Systematic review and dose-response meta-analysis of prospective cohort studies. BMJ.

[2] Koutsos A., Tuohy K.M., Lovegrove J.A. (2015). Apples and cardiovascular health-is the gut microbiota a core consideration?. Nutrients.

[3] Gerhauser C. (2008). Cancer chemopreventive potential of apples, apple juice, and apple components. Planta Med..

[4] Monagas M., Urpi-Sarda M., Sanchez-Patan F., Llorach R., Garrido I., Gomez-Cordoves C., Andres-Lacueva C., Bartolome B. (2010). Insights into the metabolism and microbial biotransformation of dietary flavan-3-ols and the bioactivity of their metabolites. Food Funct..

[5] Ozdal T., Sela D.A., Xiao J.B., Boyacioglu D., Chen F., Capanoglu E. (2016). The reciprocal interactions between polyphenols and gut microbiota and effects on bioaccessibility. Nutrients.

[6] Olano-Martin E., Gibson G.R., Rastall R.A. (2002). Comparison of the in vitro bifidogenic properties of pectins and pectic-oligosaccharides. J. Appl. Microbiol..

[7] Chen J., Liang R.-H., Liu W., Li T., Liu C.-M., Wu S.-S., Wang Z.-J. (2013). Pectic-oligosaccharides prepared by dynamic high-pressure nnicrofluidization and their in vitro fermentation properties. Carbohydr. Polym..

[8] Chung W.S.F., Walker A.W., Louis P., Parkhill J., Vermeiren J., Bosscher D., Duncan S.H., Flint H.J. (2016). Modulation of the human gut microbiota by dietary fibres occurs at the species level. BMC Biol..

[9] Vrhovsek U., Rigo A., Tonon D., Mattivi F. (2004). Quantitation of polyphenols in different apple varieties. J. Agric. Food Chem..

[10] Bazzocco S., Mattila I., Guyot S., Renard C.M.G.C., Aura A.-M. (2008). Factors affecting the conversion of apple polyphenols to phenolic acids and fruit matrix to short-chain fatty acids by human faecal microbiota in vitro. Eur. J. Nutr..

[11] Sembries S., Dongowski G., Jacobasch G., Mehrlander K., Will F., Dietrich H. (2003). Effects of dietary fibre-rich juice colloids from apple pomace extraction juices on intestinal fermentation products and microbiota in rats. Br. J. Nutr..

[12] Sembries S., Dongowski G., Mehrlaender K., Will F., Dietrich H. (2006). Physiological effects of extraction juices from apple, grape, and red beet pomaces in rats. J. Agric. Food Chem..

[13] Licht T.R., Hansen M., Bergstrom A., Poulsen M., Krath B.N., Markowski J., Dragsted L.O., Wilcks A. (2010). Effects of apples and specific apple components on the cecal environment of conventional rats: Role of apple pectin. BMC Microbiol..

[14] Jiang T., Gao X., Wu C., Tian F., Lei Q., Bi J., Xie B., Wang H.Y., Chen S., Wang X. (2016). Apple-derived pectin modulates gut microbiota, improves gut barrier function, and attenuates metabolic endotoxemia in rats with diet-induced obesity. Nutrients.

[15] Masumoto S., Terao A., Yamamoto Y., Mukai T., Miura T., Shoji T. (2016). Non-absorbable apple procyanidins prevent obesity associated with gut microbial and metabolomic changes. Sci. Rep..

[16] Aprikian O., Duclos V., Guyot S., Besson C., Manach C., Bernalier A., Morand C., Remesy C., Demigne C. (2003). Apple pectin and a polyphenol-rich apple concentrate are more effective together than separately on cecal fermentations and plasma lipids in rats. J. Nutr..

[17] Shinohara K., Ohashi Y., Kawasumi K., Terada A., Fujisawa T. (2010). Effect of apple intake on fecal microbiota and metabolites in humans. Anaerobe.

[18] Ravn-Haren G., Dragsted L.O., Buch-Andersen T., Jensen E.N., Jensen R.I., Nemeth-Balogh M., Paulovicsova B., Bergstrom A., Wilcks A., Licht T.R. (2012). Intake of whole apples or clear apple juice has contrasting effects on plasma lipids in healthy volunteers. Eur. J. Nutr..

[19] Vrhovsek U., Masuero D., Gasperotti M., Franceschi P., Caputi L., Viola R., Mattivi F. (2012). A versatile targeted metabolomics method for the rapid quantification of multiple classes of phenolics in fruits and beverages. J. Agric. Food Chem..

[20] Gasperotti M., Masuero D., Guella G., Mattivi F., Vrhovsek U. (2014). Development of a targeted method for twenty-three metabolites related to polyphenol gut microbial metabolism in biological samples, using spe and uhplc-esi-ms/ms. Talanta.

[21] Mandalari G., Faulks R.M., Rich G.T., Lo Turco V., Picout D.R., Lo Curto R.B., Bisignano G., Dugo P., Dugo G., Waldron K.W. (2008). Release of protein, lipid, and vitamin e from almond seeds during digestion. J. Agric. Food Chem..

[22] Sanchez-Patan F., Cueva C., Monagas M., Walton G.E., Gibson G.R., Quintanilla-Lopez J.E., Lebron-Aguilar R., Martin-Alvarez P.J., Moreno-Arribas M.V., Bartolome B. (2012). In vitro fermentation of a red wine extract by human gut microbiota: Changes in microbial groups and formation of phenolic metabolites. J. Agric. Food Chem..

[23] Caporaso J.G., Kuczynski J., Stombaugh J., Bittinger K., Bushman F.D., Costello E.K., Fierer N., Pena A.G., Goodrich J.K., Gordon J.I. (2010). Qiime allows analysis of high-throughput community sequencing data. Nat. Methods.

[24] Aronesty E. (2013). Comparison of sequencing utilitiy programs. Open Bioinform. J..

[25] Edgar R.C. (2010). Search and clustering orders of magnitude faster than blast. Bioinformatics.

[26] Langendijk P.S., Schut F., Jansen G.J., Raangs G.C., Kamphuis G.R., Wilkinson M.H.F., Welling G.W. (1995). Quantitative fluorescence in-situ hybridization of *Bifidobacterium* spp. with genus-specific 16s ribosomal-rna-targeted probes and its application in fecal samples. Appl. Environ. Microbiol..

[27] Hold G.L., Schwiertz A., Aminov R.I., Blaut M., Flint H.J. (2003). Oligonucleotide probes that detect quantitatively significant groups of butyrate-producing bacteria in human feces. Appl. Environ. Microbiol..

[28] Daims H., Stoecker K., Wagner M., Osborn A.M., Smith C.J. (2005). Fluorescence In Situ Hybridization for the detection of prokaryotes. Molecular Microbial Ecology.

[29] Zhao G.H., Nyman M., Jonsson J.A. (2006). Rapid determination of short-chain fatty acids in colonic contents and faeces of humans and rats by acidified water-extraction and direct-injection gas chromatography. Biomed. Chromatogr..

[30] Yang J., Martinez I., Walter J., Keshavarzian A., Rose D.J. (2013). In vitro characterization of the impact of selected dietary fibers on fecal microbiota composition and short chain fatty acid production. Anaerobe.

[31] Martinez I., Kim J., Duffy P.R., Schlegel V.L., Walter J. (2010). Resistant starches types 2 and 4 have differential effects on the composition of the fecal microbiota in human subjects. PLoS ONE.

[32] Li Z.P., Henning S.M., Lee R.P., Lu Q.Y., Summanen P.H., Thames G., Corbett K., Downes J., Tseng C.H., Finegold S.M. (2015). Pomegranate extract induces ellagitannin metabolite formation and changes stool microbiota in healthy volunteers. Food Funct..

[33] Lacombe A., Li R.W., Klimis-Zacas D., Kristo A.S., Tadepalli S., Krauss E., Young R., Wu V.C.H. (2013). Lowbush wild blueberries have the potential to modify gut microbiota and xenobiotic metabolism in the rat colon. PLoS ONE.

[34] Rossi M., Corradini C., Amaretti A., Nicolini M., Pompei A., Zanoni S., Matteuzzi D. (2005). Fermentation of fructooligosaccharides and inulin by bifidobacteria: A comparative study of pure and fecal cultures. Appl. Environ. Microbiol..

[35] Sokol H., Pigneur B., Watterlot L., Lakhdari O., Bermudez-Humaran L.G., Gratadoux J.-J., Blugeon S., Bridonneau C., Furet J.-P., Corthier G. (2008). Faecalibacterium prausnitzii is an anti-inflammatory commensal bacterium identified by gut microbiota analysis of crohn disease patients. Proc. Natl. Acad. Sci. USA.

[36] Machiels K., Joossens M., Sabino J., De Preter V., Arijs I., Eeckhaut V., Ballet V., Claes K., Van Immerseel F., Verbeke K. (2014). A decrease of the butyrate-producing species roseburia hominis and faecalibacterium prausnitzii defines dysbiosis in patients with ulcerative colitis. Gut.

[37] Pryde S.E., Duncan S.H., Hold G.L., Stewart C.S., Flint H.J. (2002). The microbiology of butyrate formation in the human colon. FEMS Microbiol. Lett..

[38] Lopez-Siles M., Khan T.M., Duncan S.H., Harmsen H.J.M., Garcia-Gil L.J., Flint H.J. (2012). Cultured representatives of two major phylogroups of human colonic faecalibacterium prausnitzii can utilize pectin, uronic acids, and host-derived substrates for growth. Appl. Environ. Microbiol..

[39] Waldecker M., Kautenburger T., Daumann H., Busch C., Schrenk D. (2008). Inhibition of histone-deacetylase activity by short-chain fatty acids and some polyphenol metabolites formed in the colon. J. Nutr. Biochem..

[40] Dolara P., Luceri C., de Filippo C., Femia A.P., Giovannelli L., Caderni G., Cecchini C., Silvi S., Orpianesi C., Cresci A. (2005). Red wine polyphenols influence carcinogenesis, intestinal microflora, oxidative damage and gene expression profiles of colonic mucosa in f344 rats. Mutat. Res.-Fundam. Mol. Mech. Mutag..

[41] Massot-Cladera M., Perez-Berezo T., Franch A., Castell M., Perez-Cano F.J. (2012). Cocoa modulatory effect on rat faecal microbiota and colonic crosstalk. Arch. Biochem. Biophys..

[42] Kemperman R.A., Gross G., Mondot S., Possemiers S., Marzorati M., van de Wiele T., Dore J., Vaughan E.E. (2013). Impact of polyphenols from black tea and red wine/grape juice on a gut model microbiome. Food Res. Int..

[43] Eid N., Enani S., Walton G., Corona G., Costabile A., Gibson G., Rowland I., Spencer J.P. (2014). The impact of date palm fruits and their component polyphenols, on gut microbial ecology, bacterial metabolites and colon cancer cell proliferation. J. Nutr. Sci..

[44] Smith A.H., Mackie R.I. (2004). Effect of condensed tannins on bacterial diversity and metabolic activity in the rat gastrointestinal tract. Appl. Environ. Microbiol..

[45] Isabel Queipo-Ortuno M., Boto-Ordonez M., Murri M., Miguel Gomez-Zumaquero J., Clemente-Postigo M., Estruch R., Cardona Diaz F., Andres-Lacueva C., Tinahones F.J. (2012). Influence of red wine polyphenols and ethanol on the gut microbiota ecology and biochemical biomarkers. Am. J. Clin. Nutr..

[46] Lyte M., Chapel A., Lyte J.M., Ai Y.F., Proctor A., Jane J.L., Phillips G.J. (2016). Resistant starch alters the microbiota-gut brain axis: Implications for dietary modulation of behavior. PLoS ONE.

[47] Walker A.W., Ince J., Duncan S.H., Webster L.M., Holtrop G., Ze X.L., Brown D., Stares M.D., Scott P., Bergerat A. (2011). Dominant and diet-responsive groups of bacteria within the human colonic microbiota. ISME J..

[48] Salonen A., Lahti L., Salojarvi J., Holtrop G., Korpela K., Duncan S.H., Date P., Farquharson F., Johnstone A.M., Lobley G.E. (2014). Impact of diet and individual variation on intestinal microbiota composition and fermentation products in obese men. ISME J..

[49] Walker A.W., Duncan S.H., McWilliam Leitch E.C., Child M.W., Flint H.J. (2005). Ph and peptide supply can radically alter bacterial populations and short-chain fatty acid ratios within microbial communities from the human colon. Appl. Environ. Microbiol..

[50] Zambell K.L., Fitch M.D., Fleming S.E. (2003). Acetate and butyrate are the major substrates for de novo lipogenesis in rat colonic epithelial cells. J. Nutr..

[51] Onumpai C., Kolida S., Bonnin E., Rastall R.A. (2011). Microbial utilization and selectivity of pectin fractions with various structures. Appl. Environ. Microbiol..

[52] Duncan S.H., Barcenilla A., Stewart C.S., Pryde S.E., Flint H.J. (2002). Acetate utilization and butyryl coenzyme a (coa): Acetate-coa transferase in butyrate-producing bacteria from the human large intestine. Appl. Environ. Microbiol..

[53] Rios-Covian D., Gueimonde M., Duncan S.H., Flint H.J., de los Reyes-Gavilan C.G. (2015). Enhanced butyrate formation by cross-feeding between faecalibacterium prausnitzii and bifidobacterium adolescentis. FEMS Microbiol. Lett..

[54] Cheng H.H., Lai M.H. (2000). Fermentation of resistant rice starch produces propionate reducing serum and hepatic cholesterol in rats. J. Nutr..

[55] Velderrain-Rodriguez G.R., Palafox-Carlos H., Wall-Medrano A., Ayala-Zavala J.F., Chen C.Y.O., Robles-Sanchez M., Astiazaran-Garcia H., Alvarez-Parrilla E., Gonzalez-Aguilar G.A. (2014). Phenolic compounds: Their journey after intake. Food Funct..

[56] Larrosa M., Luceri C., Vivoli E., Pagliuca C., Lodovici M., Moneti G., Dolara P. (2009). Polyphenol metabolites from colonic microbiota exert anti-inflammatory activity on different inflammation models. Mol. Nutr. Food Res..

[57] Appeldoorn M.M., Vincken J.P., Aura A.M., Hollman P.C.H., Gruppen H. (2009). Procyanidin dimers are metabolized by human microbiota with 2-(3,4-dihydroxyphenyl)acetic acid and 5-(3,4-dihydroxyphenyl)-gamma-valerolactone as the major metabolites. J. Agric. Food Chem..

[58] Gomez-Ruiz J.A., Leake D.S., Ames J.M. (2007). In vitro antioxidant activity of coffee compounds and their metabolites. J. Agric. Food Chem..

[59] Cueva C., Sanchez-Patan F., Monagas M., Walton G.E., Gibson G.R., Martin-Alvarez P.J., Bartolome B., Moreno-Arribas M.V. (2013). In vitro fermentation of grape seed flavan-3-ol fractions by human faecal microbiota: Changes in microbial groups and phenolic metabolites. FEMS Microbiol. Ecol..

[60] Selma M.V., Espin J.C., Tomas-Barberan F.A. (2009). Interaction between phenolics and gut microbiota: Role in human health. J. Agric. Food Chem..

[61] Dall’Asta M., Calani L., Tedeschi M., Jechiu L., Brighenti F., Del Rio D. (2012). Identification of microbial metabolites derived from in vitro fecal fermentation of different polyphenolic food sources. Nutrition.

[62] Deprez S., Brezillon C., Rabot S., Philippe C., Mila I., Lapierre C., Scalbert A. (2000). Polymeric proanthocyanidins are catabolized by human colonic microflora into low-molecular-weight phenolic acids. J. Nutr..

[63] Rios L.Y., Gonthier M.P., Remesy C., Mila L., Lapierre C., Lazarus S.A., Williamson G., Scalbert A. (2003). Chocolate intake increases urinary excretion of polyphenol-derived phenolic acids in healthy human subjects. Am. J. Clin. Nutr..

[64] Williams C.F., Walton G.E., Jiang L., Plummer S., Garaiova I., Gibson G.R., Doyle M.P., Klaenhammer T.R. (2015). Comparative analysis of intestinal tract models. Annual Review of Food Science and Technology.

[65] Johnson L.P., Walton G.E., Psichas A., Frost G.S., Gibson G.R., Barraclough T.G. (2015). Prebiotics modulate the effects of antibiotics on gut microbial diversity and functioning in vitro. Nutrients.

[66] Rodrigues D., Walton G., Sousa S., Rocha-Santos T.A.P., Duarte A.C., Freitas A.C., Gomes A.M.P. (2016). In vitro fermentation and prebiotic potential of selected extracts from seaweeds and mushrooms. LWT -Food Sci. Technol..

[67] Blatchford P., Stoklosinski H., Walton G., Swann J., Gibson G., Gearry R., Ansell J. (2015). Kiwifruit fermentation drives positive gut microbial and metabolic changes irrespective of initial microbiota composition. Bioact. Carbohydr. Diet. Fibre.

[68] Walton G.E., van den Heuvel E., Kosters M.H.W., Rastall R.A., Tuohy K.M., Gibson G.R. (2012). A randomised crossover study investigating the effects of galacto-oligosaccharides on the faecal microbiota in men and women over 50 years of age. Br. J. Nutr..

