# Supplementary material for: Effects of Commercial Apple Varieties on Human Gut Microbiota Composition and Metabolic Output Using an In Vitro Colonic Model
Source: Nutrients. 2017 May 24;9(6):533. doi: 10.3390/nu9060533 (PMC5490512; doi:10.3390/nu9060533)
Supplement: Supplementary file 1 [file nutrients-09-00533-s001.zip › nutrients-180000-supplementary.pdf]

Supplementary File

Table S1. Composition analysis of Renetta Canada, Golden Delicious and Pink Lady. *

**Components**	**Renetta Canada**	**Golden Delicious**	**Pink Lady**
Energy (KJ)/100g	274	297	267
Energy (Kcal)/100g	65	70	63
Fat (Weibull Stoldt) (g/100g)	0.2	0.3	0.2
Carbohydrate total (g/100g)	16.8	17.8	16.2
Total sugar (g/100g)	13.3	12.9	13.0
Glucose (g/100g)	1.5	0.8	1.5
Fructose (g/100g)	6.4	5.6	6.4
Sucrose (g/100g)	5.4	6.5	5.1
Protein (Kjeldahl) (g/100g)	0.3	0.3	0.2
Moisture (Vac at 70°C) (g/100g)	82.5	81.3	83.0
Organic acids			
Ascorbic acid	0.6	0.4	0.9
Malic acid	902	478	729

* For each apple variety a mixture of three fresh whole apples was analyzed.

A

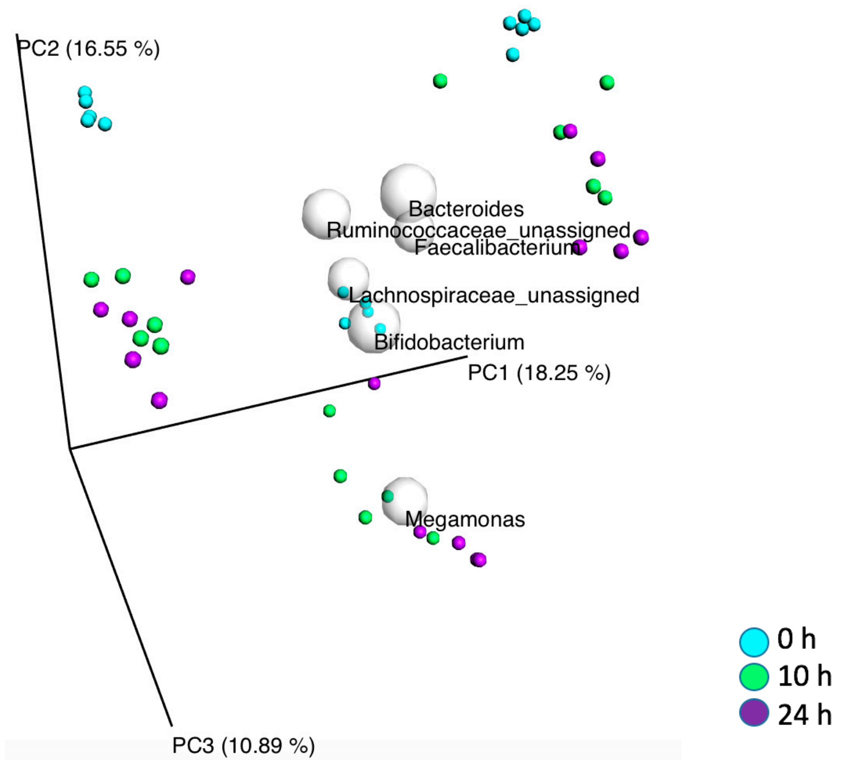

B

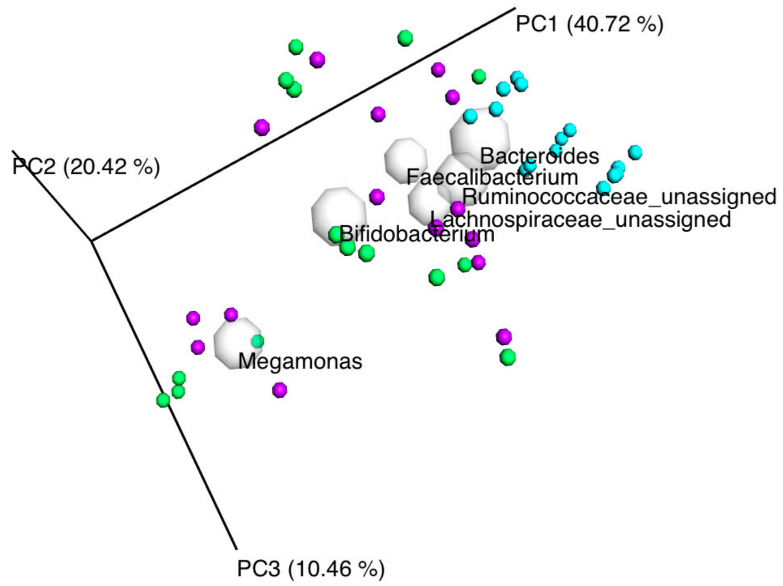

Figure S1. Principal coordinate analysis (PCoA) plots of 16S rRNA gene profiles based on (A) unweighted (qualitative) and (B) weighted (quantitative) phylogenetic UniFrac distance matrices calculated from a rarefied OTU table (11708 reads per sample) showing a clustering between time (ANOSIM and ADONIS test, P=0.01 and P=0.001, respectively) for the whole data set (24-hour *in vitro* batch culture fermentations inoculated with human feces (n=3 healthy donors) and administrated with inulin, cellulose, Renetta Canada, Golden Delicious and Pink Lady as the substrates/treatments). Samples were analyzed at 0, 10 and 24 h. Each color represents a different time point. The gray spherical coordinates indicate taxonomic vectors of the 6 most prevalent taxa at the genus level. The size of each sphere is proportional to the mean relative abundance and approximates the causing variance throughout the plotted samples.

A

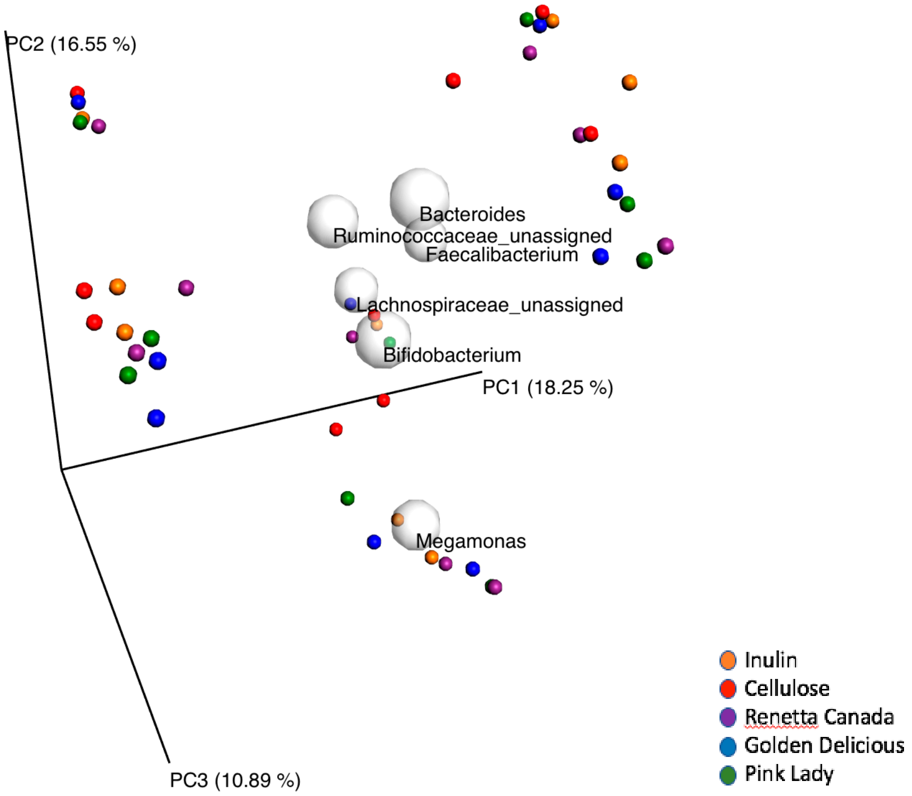

B

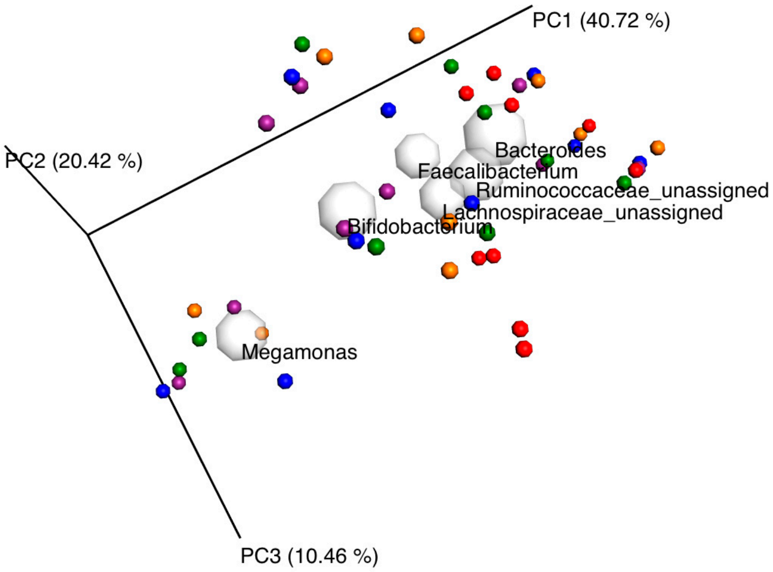

Figure S2. Principal coordinate analysis (PCoA) plots of 16S rRNA gene profiles based on (A) unweighted (qualitative) and (B) weighted (quantitative) phylogenetic UniFrac distance matrices calculated from a rarefied OTU table (11708 reads per sample) and colored according to treatment (24-hour *in vitro* batch culture fermentations inoculated with human feces (n=3 healthy donors) and administrated with inulin, cellulose, Renetta Canada, Golden Delicious and Pink Lady as the substrates/treatments). Samples were analyzed at 0, 10 and 24 h. Each color represents a different treatment. The gray spherical coordinates indicate taxonomic vectors of the 6 most prevalent taxa at the genus level. The size of each sphere is proportional to the mean relative abundance and approximates the causing variance throughout the plotted samples.

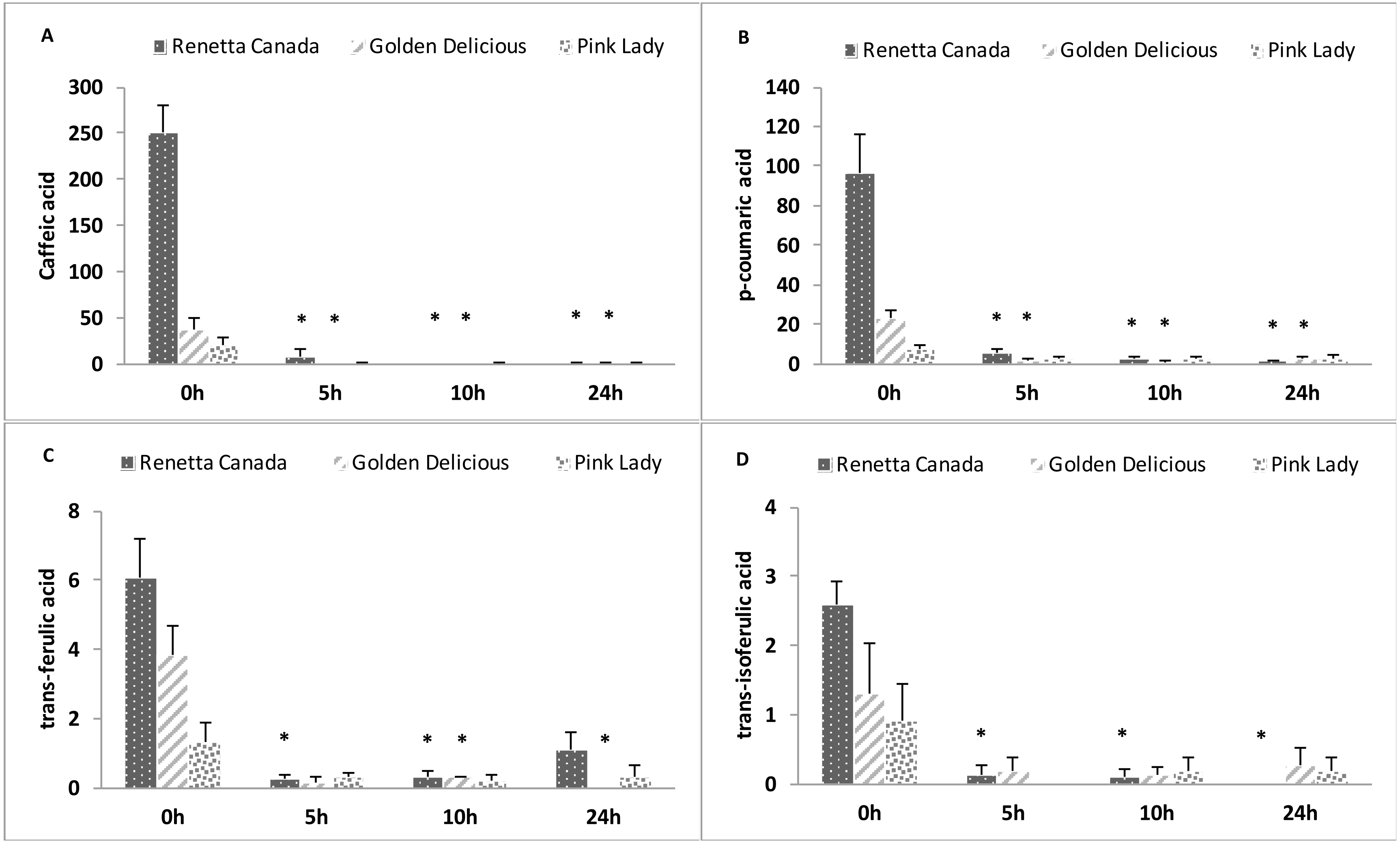

Figure S3. Changes in cinnamic acid derivatives (**A**, Caffeic acid; **B**, p-coumaric acid; **C**, t-ferulic acid and **D,** trans-isoferulic acid) throughout 24-hour *in vitro* batch culture fermentations inoculated with human feces (n=3 healthy donors) and administrated with Renetta Canada, Golden Delicious and Pink Lady as the substrates (treatments). Samples were collected at 0, 5, 10 and 24 h. Results are expressed as ng/ml of batch culture medium and values are mean ± SEM of the three fermentations. * Significant differences (P<0.05) from the 0 h time point within the same treatment

Table S2. Changes in bacterial taxa throughout 24-hour *in vitro* batch culture fermentations inoculated with human feces (n=3 healthy donors) and administrated with inulin, cellulose, Renetta Canada, Golden Delicious and Pink Lady as the substrates.

**Bacterial Taxonomy**	**Time Point**	**Inulin (%)**	***p* (*p*-FDR)[a]**	**Cellulose (%)**	***p* (*p*-FDR)[a]**	**Renetta Canada (%)**	***p* (*p*-FDR)[a]**	**Golden Delicious (%)**	***p* (*p*-FDR)[a]**	**Pink Lady (%)**	***p* (*p*-FDR)[a]**
p_Firmicutes f_*Lachnospiraceae* g_*Blautia*	0h	3.4 ± 1.1	0.880 (0.985)	3.6 ± 1.1	0.090 (0.548)	4.8 ± 0.43	**0.022** (0.211)	4.2 ± 0.32	0.082 (0.423)	4.9 ± 0.9	**0.009** (0.121)
	10h	3.8 ± 0.9		2.5 ± 1.1		1.9 ± 1.2		2.4 ± 1.2		2.2 ± 1.1	
	24h	3.3 ± 2		1.2 ± 0.37		1.5 ± 0.54*		1.8 ± 1.1		1.1 ± 0.4*	
p_Firmicutes; f_*Lachnospiraceae* g_*Coprococcus*	0h	5.9 ±1.6	0.820 (0.985)	5.7 ± 0.87	**0.001 (0.034)**	5.6 ± 1.6	0.873 (0.985)	5.2 ± 1.2	0.806 (0.985)	5.9 ± 1.2	0.441 (0.951)
	10h	5.5 ± 2		4.4 ± 0.89*		6 ± 2.3		5.6 ± 2.6		3.2 ± 1.2	
	24h	4.3 ± 1.3		4 ± 0.91*		4.4 ± 1.6		7.3 ± 3.5		3.7 ± 1.3	
p_Firmicutes f_*Lachnospiraceae* g_*Dorea*	0h	0.87 ± 0.16	0.163 (0.683)	0.93 ± 0.26	**0.015** (0.201)	1.1 ± 0.22	0.068 (0.350)	0.9 ± 0.18	0.410 (0.985)	1.3 ± 0.4	0.128 (0.504)
	10h	6.9 ± 3.6		4.8 ± 0.93*		3.5 ± 0.94		3.6 ± 1.9		4.3 ± 2.3	
	24h	3.4 ± 1.5		5.6 ± 1.3		2.3 ± 0.11		2.7 ± 0.96		5.4 ± 0.39	
p_Firmicutes; f_*Lachnospiraceae* g_*Roseburia*	0h	0.38 ± 0.05	**0.020** (0.268)	0.79 ± 0.30	0.084 (0.563)	0.48 ± 0.11	**0.026** (0.218)	0.53 ± 0.08	0.156 (0.615)	0.47 ± 0.04	**0.049** (0.298)
	10h	0.12 ± 0.06		0.06 ± 0.02		0.03 ± 0.01		0.03 ± 0.01		0.06 ± 0.02*	
	24h	0.04 ± 0.01*		0.08 ± 0.07		0.06 ± 0.04		0.25 ± 0.21		0.17 ± 0.13	
p_Firmicutes f_*Lachnospiraceae* g_unassigned	0h	5.6 ± 0.17	0.342 (0.979)	6 ± 0.81	0.059 (0.494)	6.1 ± 0.21	**0.030** (0.223)	5.6 ± 0.35	0.162 (0.603)	6.5 ± 0.76	**0.003** (0.067)
	10h	4.3 ± 1.3		9.1 ± 0.94		2.6 ± 0.66		5 ± 2.1		2.9 ± 1.2	
	24h	6.5 ± 0.8		10 ± 1.2		9.3 ± 1.9		10 ± 1.5		10.3 ± 1.3*	
p_Firmicutes; f_*Ruminococcaceae* g_*Faecalibacterium*	0h	5.2 ± 1.6	**0.035** (0.335)	5.5 ± 2	0.689 (0.985)	5.8 ± 2.5	**0.019** (0.255)	5 ± 1.7	0.755 (0.985)	5.8 ± 2.7	0.386 (0.951)
	10h	6.1 ± 2.6		4.5 ± 0.99		4.9 ± 2.9		5.1 ± 2.7		2.8 ± 0.93	
	24h	17 ± 3.7		4.6 ±2.2		16 ± 5.5		5.9 ± 1.9		5.4 ± 1.4	
p_Firmicutes; f_*Ruminococcaceae* g_*Oscillospira*	0h	0.9 ± 0.2	0.667 (0.985)	0.91 ± 0.19	**0.002** (0.054)	0.98 ± 0.31	0.218 (0.730)	0.96 ± 0.29	**0.040** (0.335)	0.94 ± 0.3	**0.019** (0.182)
	10h	0.66 ± 0.10		1 ± 0.07		0.32 ± 0.03		0.30 ± 0.14		0.23 ± 0.07	
	24h	1 ± 0.58		2.5 ± 0.11*		1 ± 0.49		1.1 ± 0.35		1.4 ± 0.28*	

p_Firmicutes	0h	4 ± 0.96	**0.015** (0.251)	4.9 ± 1.4	0.100 (0.558)	4.7 ± 1.1	**0.011** (0.246)	4.3 ± 1	**0.013** (0.290)	4.5 ± 1.2	**0.021** (0.176)
f_*Ruminococcaceae*	10h	1.4 ± 0.35		1.8 ± 0.49		0.63 ± 0.43*		0.41 ± 0.18		0.45 ± 0.17	
g_*Ruminococcus*	24h	0.74 ± 0.11		1.2 ± 0.62		0.39 ± 0.12		0.34 ± 0.16		0.22 ± 0.13	
p_Firmicutes;	0h	0.31 ± 0.04	**0.009** (0.201)	0.29 ± 0.06	0.263 (0.985)	0.30 ± 0.06	**0.022** (0.246)	0.24 ± 0.08	0.122 (0.545)	0.29 ± 0.02	**0.000 (0.007)**
f_[*Mogibacteriaceae*]	10h	0.15 ± 0.01		0.14 ± 0.00		0.08 ± 0.02		0.05 ± 0.01		0.06 ± 0.01*	
g_unassigned	24h	0.06 ± 0.01*		0.16 ± 0.06		0.08 ± 0.02		0.08 ± 0.02		0.07 ± 0.03*	
p_Firmicutes;	0h	4.3 ± 0.73	**0.021** (0.235)	4.9 ± 0.69	**0.043** (0.412)	4.6 ± 0.61	**0.001 (0.040)**	4.8 ± 0.78	**0.002** (0.084)	4.1 ± 0.63	**0.004** (0.067)
o_Clostridiales;	10h	1.8 ± 0.09		2.8 ± 0.33*		0.82 ± 0.24*		0.76 ± 0.16*		0.81 ± 0.12*	
f_g_unclassified	24h	1.2 ± 0.27		2.5 ±0.58		0.36 ± 0.05*		0.75 ± 0.19*		0.86 ± 0.19*	
p_Firmicutes;	0h	2.7 ± 0.18	**0.001 (0.050)**	2.7 ± 0.21	**0.007** (0.117)	2.7 ± 0.44	**0.002** (0.067)	2.5 ± 0.25	**0.001 (0.040)**	3.3 ± 0.78	**0.045** (0.302)
o_Clostridiales;	10h	0.68 ± 0.12*		1.4 ± 0.23*		0.47 ± 0.10*		0.33 ± 0.03*		0.59 ± 0.27	
Other;Other	24h	0.71 ± 0.29*		1.4 ± 0.41*		0.68 ± 0.19*		0.34 ± 0.05*		0.81 ± 0.65	
p_Firmicutes;	0h	0.04 ± 0.02	0.263 (0.979)	0.06 ± 0	0.366 (0.985)	0.05 ± 0.01	0.272 (0.868)	0.03 ± 0.01	0.133 (0.557)	0.04 ± 0.00	**0.001 (0.034)**
f_*Turicibacteraceae*	10h	0.02 ± 0.01		0.03 ± 0.02		0.01 ± 0.01		0.01 ± 0.01		0.01 ± 0.01*	
g_*Turicibacter*	24h	0.02 ± 0.01		0.03 ± 0.02		0.02 ± 0.02		0.00 ± 0.00		0.01 ± 0.01*	
p_Bacteroidetes	0h	18 ± 4.2	**0.006** (0.201)	15.1 ± 3.1	0.349 (0.985)	17.5 ± 3.2	**0.016** (0.268)	17.2 ± 4.8	**0.031** (0.346)	14 ± 2.3	0.423 (0.951)
f_*Bacteroidaceae*	10h	7.2 ± 3.8*		12.5 ± 6		2.3 ± 1.02*		2.8 ± 1.1		6.2 ± 4	
g_*Bacteroides*	24h	3.6 ± 1.4*		11.6 ± 3.8		3 ± 1.46		9.5 ± 3.6		12.6 ± 8.9	
p_Bacteroidetes	0h	0.32 ± 0.09	0.063 (0.422)	0.37 ± 0.13	0.875 (0.985)	0.33 ± 0.12	0.095 (0.398)	0.30 ± 0.15	0.535 (0.985)	0.26 ± 0.08	**0.016** (0.179)
f_[*Odoribacteraceae*]	10h	0.14 ± 0.05		0.33 ± 0.11		0.05 ± 0.04		0.10 ± 0.04		0.19 ± 0.09*	
g_*Odoribacter*	24h	0.06 ± 0.02		0.41 ± 0.24		0.02 ± 0.02		0.15 ± 0.13		0.07 ± 0.04*	
p_Bacteroidetes	0h	2.8 ± 1.2	0.116 (0.518)	2.2 ± 0.88	0.125 (0.598)	2.1 ± 0.76	**0.050** (0.279)	2.3 ± 0.76	**0.043** (0.288)	2.4 ± 0.9	0.060 (0.287)
f_*Rikenellaceae*	10h	0.86 ± 0.29		1.4 ± 0.54		0.6 ± 0.22		0.48 ± 0.18		0.97 ± 0.43	
g_unassigned	24h	0.91 ± 0.45		1.1 ±0.26		0.57 ± 0.28		0.70 ± 0.46		0.59 ± 0.3	
p_Actinobacteria	0h	2.5 ± 0.28	0.569 (0.979)	2.5 ± 0.1	0.376 (0.985)	3.5 ± 0.1	**0.049** (0.305)	3.3 ± 0.61	**0.016** (0.268)	3.8 ± 0.86	0.069 (0.308)
f_*Bifidobacteriaceae*	10h	4.7 ± 2.4		2.8 ± 0.21		24.3 ± 8.7		19.4 ± 4.2		16.2 ± 2.1	
g_*Bifidobacterium*	24h	6.3 ± 4.6		2.6 ± 0.21		14.9 ± 2.5*		15.2 ± 2.6		10.4 ± 3.5	
	0h	2 ± 0.2		1.9 ± 0.14		1.7 ± 0.24		1.8 ± 0.07		2.1 ± 0.14	

p_Actinobacteria f_*Coriobacteriaceae* g_*Collinsella*	10h	8.6 ± 2.6	**0.050** (0.394)	3.2 ± 0.06*	**0.001** **(0.040)**	5.6 ± 1.8	0.097 (0.382)	5.9 ± 1.5	**0.050** (0.274)	5.5 ± 1.4	0.052 (0.290)
	24h	7 ± 1.6		3.8 ± 0.17*		4.7 ± 1.1		5.2 ± 0.95		8.4 ± 2	
p_Actinobacteria f_*Coriobacteriaceae* g_unassigned	0h	0.62 ± 0.16	0.250 (0.985)	0.61 ± 0.18	0.849 (0.985)	0.55 ± 0.09	0.693 (0.985)	0.65 ± 0.17	**0.042** (0.313)	0.82 ± 0.36	0.352 (0.951)
	10h	0.60 ± 0.18		0.5 ± 0.09		0.50 ± 0.17		0.38 ± 0.13		0.45 ± 0.17	
	24h	0.33 ± 0.02		0.57 ± 0.11		0.44 ± 0.16		0.31 ± 0.06		0.44 ± 0.01	
p_Proteobacteria f_*Alcaligenaceae* g_*Sutterella*	0h	1.1 ± 0.04	0.104 (0.498)	1 ± 0.14	**0.035** (0.391)	1.2 ± 0.18	0.085 (0.380)	1.4 ± 0.2	0.051 (0.285)	1.1 ± 0.2	0.165 (0.582)
	10h	4.8 ± 1.9		7.2 ± 2.6		6.3 ± 2.2		6.4 ± 2.2		8.9 ± 4.6	
	24h	4.5 ± 1.4		9.6 ± 2.6		5.9 ±1.9		6.1 ± 1.4		5.2 ± 2.5	
p_Proteobacteria f_*Brucellaceae* g_*Ochrobactrum*	0h	0.05 ± 0.02	0.626 (0.979)	0.06 ± 0.02	0.362 (0.985)	0.06 ± 0.02	0.396 (0.985)	0.09 ± 0.03	**0.031** (0.297)	0.09 ± 0.01	**0.045** (0.335)
	10h	0.03 ± 0.02		0.03 ± 0.02		0.04 ± 0.02		0.03 ± 0.02		0.03 ± 0.01*	
	24h	0.03 ± 0.02		0.02 ± 0.01		0.03 ± 0.02		0.02 ± 0.01		0.03 ± 0.02	
p_Proteobacteria f_*Desulfovibrionaceae* g_*Bilophila*	0h	0.13 ± 0.02	**0.050** (0.372)	0.06 ± 0.01	0.102 (0.526)	0.12 ± 0.05	0.074 (0.354)	0.10 ± 0.01	**0.031** (0.415)	0.15 ± 0.00	0.284 (0.951)
	10h	0.33 ± 0.03*		1.7 ± 0.65		0.17 ± 0.01		0.24 ± 0.08		0.26 ± 0.05	
	24h	0.84 ± 0.25		3.3 ±1.4		0.74 ± 0.27		1.5 ± 0.44		1.5 ± 0.93	

Samples were analyzed at 0, 10 and 24 h. Results are expressed as relative abundance (%) and values are mean ± SEM of the three fermentations. [a] *p*=raw/unadjusted *p* values, *p*-FDR=corrected with false discovery rate method for 67 taxa. * Significant differences ($p < 0.05$) from the 0 h time point within the same treatment. Brackets indicate suggested but not verified names. p: phylum; f: family; g: genus.

Table S3. Multiple Reaction Monitoring (MRM) conditions of precursor polyphenols and polyphenol microbial metabolites.

Compound	RT (min)	ESI mode	Precursor ion (m/z)	Quantifier Product ion (m/z)	Qualifier Product ion (m/z)
**Precursor polyphenols**					
**Flavan-3-ols**					
Catechin	2.80	-	289	203	123
Epicatechin	3.32	-	289	203	123
Procyanidin A2	4.62	-	575	285	449
Procyanidin B1	2.40	-	577	289	425
Procyanidin B2 + B4	3.01	-	577	289	425
**Hydroxycinnamates**					
Chlorogenic acid	2.76	-	353	191	
Neochlorogenic acid	2.18	-	353	191	179
Cryptochlorogenic acid	2.87	-	353	173	179
**Flavonols**					
Quercetin	8.40	+	303	153	229
Quercetin-3-*O*-glucoside	4.50	+	465	303	229
quercetin-3-*O*-rhamnoside	5.55	+	449	303	129
Kaempferol	8.45	+	287	153	165
Isorhamnetin	8.67	+	317	153	302
Laricitrin	7.46	+	333	318	218
**Dihydrochalcones**					
Phloretin	8.23	-	273	167	123
Phlorizin	6.22	-	435	273	167
**Flavones**					
Luteolin	7.37	+	287	153	135
**Other/benzoic acid derivatives**					
Ellagic acid	4.38	-	301	145	185
Vanillin	4.07	+	153	93	125
Vanillic acid	3.23	+	169	93	65
**Polyphenol microbial metabolites**					
**Phenylacetic acid derivatives**					
3-Hydroxyphenylacetic acid	3.40	-	151	65	79
3,4-Dihydroxyphenylacetic acid	2.33	+	169		77
3,4-Dihydroxyphenylacetic acid	2.33	-	167	95	
Homovanillic acid	3.39	+	183	137	122
**Phenylpropionic acid derivatives**					
3-(3-Hydroxyphenyl)propionic acid	4.28	+	167	121	107
3-(4-Hydroxyphenyl)propionic acid	3.81	+	167	107	
3-(4-Hydroxyphenyl)propionic acid	3.81	-	165		93

Hydroferulic acid	4.20	-	195	136	121
**Benzoic acid Derivatives**					
Gallic acid	1.39	+	171	109	81
4-Hydroxybenzoic acid	2.83	+	139	77	65
Protocatechuic acid	2.10	+	155	93	65
Pyrocatechol	2.86	-	109	81	53
**Cinnamic acid Derivatives**					
Caffeic acid	3.18	+	181	145	117
p-Coumaric acid	4.01	+	165	91	119
trans-Ferulic acid	4.49	+	195	145	117
trans-Isoferulic acid	4.80	+	195	145	117

Table S4. Changes in precursor polyphenols throughout 24-hour *in vitro* batch culture fermentations inoculated with human feces (n=3 healthy donors) and administrated with inulin, cellulose, Renetta Canada, Golden Delicious and Pink Lady as the substrates.

Apple variety	0h	5h	10h	24h	*p*
**Catechin (ng/ml)**					
Renetta Canada	127.8 ± 31.3[b]	19.1 ± 11.9	9 ± 7.4*	3.7 ± 3.7*	0.005
Golden Delicious	7.1 ± 4.2[a]	0.1 ± 0.05	0.9 ± 0.5	1.1 ± 1.1	0.169
Pink Lady	0.14 ± 0.07[a]	1.3 ± 0.7	0.36 ± 0.36	0.29 ± 0.26	0.292
**Epicatechin (ng/ml)**					
Renetta Canada	449.3 ± 32.4[b]	27.6 ± 15.8*	8.1 ± 2.3*	4.9 ± 3.1*	0.000
Golden Delicious	55.7 ± 15.2[a]	4.6 ± 1.6*	0.6 ± 0.6*	2.3 ± 2.3*	0.006
Pink Lady	6.3 ± 2.9[a]	0.8 ± 0.8	1.3 ± 1.3	0.66 ± 0.66	0.119
**Procyanidin A2 (ng/ml)**					
Renetta Canada	239.1 ± 45.1[b]	58 ± 36.8	21 ± 17.9*	0.14 ± 0.14*	0.003
Golden Delicious	96.3 ± 39.5[a]	17.5 ± 8.5	3 ± 3	0.17 ± 0.17	0.030
Pink Lady	37.9 ± 7.8[a]	16.3 ± 8.2*	3.2 ± 3.2*	0.98 ± 0.98*	0.005
**Procyanidin B1 (ng/ml)**					
Renetta Canada	82.3 ± 40.4[b]	27.9 ± 25.9	1.5 ± 0.9	1.1 ± 0.6	0.088
Golden Delicious	2.3 ± 1.7[a]	0 ± 0	2.4 ± 1.7	0 ± 0	0.224
Pink Lady	1.4 ± 0.3[a]	0 ± 0	1.2 ± 1.2	0 ± 0	0.359
**Procyanidin B2 B4 (ng/ml)**					
Renetta Canada	233.7 ± 140.8	22.1 ± 19.5	2.1 ± 0.6	0.56 ± 0.44	0.132
Golden Delicious	13.4 ± 10	0.13 ± 0.13	0 ± 0	0 ± 0	0.250
Pink Lady	0.89 ± 0.41	0 ± 0	2.4 ± 2.1	0 ± 0	0.376
**Chlorogenic acid (ng/ml)**					
Renetta Canada	215.4 ±114.6	0.37 ± 0.2	0 ± 0	0 ± 0	0.088
Golden Delicious	1.3 ± 0.7	0.03 ± 0.03	0.15 ± 0.15	0.13 ± 0.13	0.160
Pink Lady	0.56 ± 0.31	0.12 ± 0.08	0 ± 0	0.15 ± 0.25	0.244
**Neochlorogenic acid (ng/ml)**					
Renetta Canada	65 ± 31.4	17.1 ± 14.4	4.2 ± 2.4	1.4 ± 1.4	0.061
Golden Delicious	10 ± 1.4	0.8 ± 0.6*	0.56 ± 0.56*	0 ± 0*	0.000
Pink Lady	6.1 ± 2.6	1.1 ± 0.2	0.32 ± 0.18	0 ± 0	0.053
**Cryptochlorogenic acid (ng/ml)**					
Renetta Canada	21.2 ± 10.8	0.08 ± 0.08	0 ± 0	0.09 ± 0.09	0.074
Golden Delicious	1.9 ± 0.7	0 ± 0	0.31 ± 0.31	0.00 ± 0.00	0.033
Pink Lady	0.6 ± 0.26	0.09 ± 0.09	0 ± 0	0.52 ± 0.41	0.377
**Quercetin (ng/ml)**					
Renetta Canada	168.9 ± 34.3	125.9 ± 124.8	64.8 ± 64.6	33.7 ± 33.3	0.273
Golden Delicious	358.1 ± 53.4	526.9 ± 522.4	430.5 ± 429.2	4.9 ± 4.9	0.573
Pink Lady	408.8 ± 102.1	449.2 ± 444.9	208.6 ± 207.1	2.2 ± 2	0.527
**Quercetin-3-Glc (ng/ml)**					
Renetta Canada	1.2 ± 0.3[a]	0.0 ± 0.0*	0 ± 0*	0.3 ± 0.16	0.006
Golden Delicious	386.1 ± 48.7[c]	0.73 ± 0.4*	0 ± 0*	0.27 ± 0.27*	0.000
Pink Lady	165.3 ± 20.6[b]	0.2 ± 0.2*	0.22 ± 0.22*	0.2 ± 0.2*	0.000
**Quercetin-3-Rha (ng/ml)**					
Renetta Canada	69.2 ± 14.7[a]	0.08 ± 0.08*	0.03 ± 0.03*	0 ± 0*	0.001
Golden Delicious	218.6 ± 34.6[b]	0.31 ± 0.16*	0.03 ± 0.03*	0.03 ± 0.03*	0.000
Pink Lady	164.5 ± 40.7[ab]	0.50 ± 0.37*	0.07 ± 0.07*	0.00 ± 0.00*	0.003
**Kaempferol (ng/ml)**					
Renetta Canada	22.8 ± 4.5	5 ± 4.2*	4.2 ± 4*	3.7 ± 3.3*	0.000
Golden Delicious	11.7 ± 2.1	6.5 ± 6.3	2.2 ± 2.2	0.1 ± 0.1	0.095
Pink Lady	11.3 ± 1.5	7.3 ± 5.7	1.3 ± 0.7	0.23 ± 0.15	0.095
**Isorhamnetin (ng/ml)**					
Renetta Canada	2.6 ± 0.4	0.85 ± 0.6*	0.6 ± 0.5*	0.69 ± 0.63*	0.000

**Golden Delicious**	4.2 ± 0.9	2.1 ± 2.1	1.3 ± 1	0 ± 0	0.077
**Pink Lady**	3.9 ± 0.9	1.9 ± 1.9	0.4 ± 0.1	0 ± 0	0.146
**Laricitrin (ng/ml)**					
**Renetta Canada**	6.7 ± 1.3	8.4 ± 7.2	5.9 ± 5.5	3.3 ± 2.9	0.684
**Golden Delicious**	22.1 ± 4.5	24.8 ± 21.2	22.8 ± 22.1	6 ± 5.8	0.614
**Pink Lady**	18.6 ± 5.9	20 ± 16.4	8.1 ± 7.3	5.9 ± 5.2	0.555
**Phloretin (ng/ml)**					
**Renetta Canada**	120.8 ± 23 [b]	614.3 ± 544.7	103.2 ± 102	0.48 ± 0.48	0.404
**Golden Delicious**	28 ± 4.2 [a]	106.3 ± 101.6	43.6 ± 43.6	0 ± 0	0.492
**Pink Lady**	4.5 ± 1 [a]	22.8 ± 21	10.9 ± 10.9	0.03 ± 0.03	0.456
**Phlorizin (ng/ml)**					
**Renetta Canada**	592.8 ± 233.2 [b]	0.64 ± 0.53	0.07 ± 0.04	0.02 ± 0.02	0.026
**Golden Delicious**	78.4 ± 29.1 [a]	0.03 ± 0.03	0 ± 0	0 ± 0	0.020
**Pink Lady**	3.2 ± 1 [a]	3.55 ± 3.51	0 ± 0	0 ± 0	0.459
**Luteolin (ng/ml)**					
**Renetta Canada**	1.2 ± 0.17	0.38 ± 0.33	0.47 ± 0.43	0.6 ± 0.6	0.292
**Golden Delicious**	0.65 ± 0.18	0.73 ± 0.73	0.38 ± 0.23	0.22 ± 0.22	0.725
**Pink Lady**	1.10 ± 0.45	0.79 ± 0.66	0.27 ± 0.14	0.09 ± 0.09	0.453
**Ellagic acid (ng/ml)**					
**Renetta Canada**	21.5 ± 3	40 ± 16.6	20.6 ± 8	18.6 ± 8	0.461
**Golden Delicious**	22.4 ± 2.1	19.8 ± 6.85	12.4 ± 6.2	20.9 ± 3.6	0.556
**Pink Lady**	21.1 ± 3.6	34.1 ± 14.1	22.7 ± 11.7	31.1 ± 9.6	0.514
**Vanillin (ng/ml)**					
**Renetta Canada**	6.8 ± 0.1 [b]	3.5 ± 1.4	2.4 ± 0.9*	2 ± 0.24*	0.008
**Golden Delicious**	5.2 ± 0.5 [a]	2.5 ± 0.8	1.9 ± 0.8*	1.3 ± 0.27*	0.004
**Pink Lady**	4.8 ± 0.5 [a]	3.4 ± 1.3	4.7 ± 2.1	2.2 ± 0.47	0.496
**Vanillic acid (ng/ml)**					
**Renetta Canada**	4.7 ± 0.9	5.5 ± 2.1	3.1 ± 1.8	2.3 ± 1.2	0.397
**Golden Delicious**	3.4 ± 0.5	2.3 ± 1.4	0.79 ± 0.58	1.8 ± 0.95	0.066
**Pink Lady**	4.2 ± 1.1	3.9 ± 1.9	1.9 ± 1.1	1.9 ± 0.90	0.323

Samples were collected at 0, 5, 10 and 24 h. Results are expressed as ng/ml of batch culture medium and values are mean ± SEM of the three fermentations. Significant differences ($p < 0.05$) between treatments at the same time point are indicated with different letters. * Significant differences ($p < 0.05$) from the 0 h time point within the same treatment.
